# Catalytic Enantioselective Construction of Carbon–Sulfur Bonds: Recent Advances and Future Perspectives

**DOI:** 10.3390/molecules31152616

**Published:** 2026-07-27

**Authors:** Michał Rachwalski

**Affiliations:** Department of Organic and Applied Chemistry, Faculty of Chemistry, University of Lodz, Tamka 12, PL-91-403 Lodz, Poland; michal.rachwalski@chemia.uni.lodz.pl; Tel.: +48-42-6355767

**Keywords:** asymmetric synthesis, carbon–sulfur bond formation, enantioselective catalysis, organocatalysis, organosulfur compounds, photoredox catalysis, sulfoximines, sulfur chemistry, sulfur stereogenicity, transition-metal catalysis

## Abstract

The catalytic enantioselective formation of carbon–sulfur (C–S) bonds is a rapidly evolving field in modern organic synthesis, driven by the importance of chiral sulfur-containing motifs in pharmaceuticals, agrochemicals, and functional materials. Despite their significance, asymmetric C–S bond-forming reactions remain challenging due to the strong coordinating ability and nucleophilicity of sulfur species, catalyst deactivation, and difficulties in controlling sulfur- or carbon-centered stereogenicity. Recent advances in transition-metal catalysis, organocatalysis, and emerging electrocatalytic strategies have enabled efficient access to diverse chiral organosulfur frameworks, including sulfides, sulfoxides, sulfilimines, sulfoximines, and sulfinamides, with high levels of enantioselectivity. These methodologies have significantly expanded the synthetic toolbox for constructing C–S bonds under mild and sustainable conditions. This review provides a concise and critical overview of catalytic enantioselective C–S bond formation, emphasizing reaction design, catalytic systems, and mechanistic aspects of enantiocontrol. Key transformations such as asymmetric allylic substitution, conjugate addition, cross-coupling, photoredox-enabled processes, and emerging electrochemical approaches are discussed. Particular attention is given to strategies for the generation and control of sulfur-centered stereogenicity. Finally, current limitations and future opportunities, including dual catalysis, late-stage functionalization, and sustainable catalytic systems, are highlighted to guide further developments in asymmetric organosulfur chemistry.

## 1. Introduction

### 1.1. Importance of C–S Bonds in Organic Chemistry

Carbon–sulfur (C–S) bonds constitute one of the most important structural motifs in modern organic chemistry. Sulfur-containing organic compounds are ubiquitous in biologically active molecules, pharmaceuticals, agrochemicals, natural products, flavors fragrances, and functional materials [1,2,3,4]. The unique physicochemical properties of sulfur arise from its variable oxidation states, high polarizability, and ability to participate in diverse non-covalent interactions. Consequently, incorporation of sulfur-containing functionalities often leads to substantial modifications of molecular reactivity, metabolic stability, lipophilicity, and biological activity.

Numerous marketed drugs contain sulfur-based functional groups, including sulfides, sulfoxides, sulfones, sulfonamides, sulfoximines, and sulfilimines. In medicinal chemistry, sulfur-containing moieties are frequently employed as bioisosteres and pharmacophoric elements that modulate target binding and pharmacokinetic properties. The growing interest in sulfur (VI) functionalities, particularly sulfoximines and sulfonimidamides, further highlights the strategic importance of sulfur chemistry in contemporary drug discovery [5,6,7].

Beyond pharmaceutical applications, organosulfur compounds play essential roles in materials science and catalysis. Sulfur-containing ligands are widely utilized in transition-metal catalysis, whereas chiral sulfur compounds have become valuable building blocks in asymmetric synthesis. Moreover, sulfur-centered functionalities are increasingly exploited in late-stage functionalization and diversity-oriented synthesis due to their unique reactivity patterns [8,9,10].

As a consequence, the development of efficient and selective methods for C–S bond formation remains a central objective in synthetic organic chemistry. Significant advances have been achieved during the last two decades, particularly through the emergence of transition-metal catalysis, organocatalysis, photocatalysis, and electrochemical approaches. These methodologies have dramatically expanded the scope of accessible sulfur-containing molecular architectures while improving functional-group tolerance and sustainability [11,12,13,14].

### 1.2. Challenges in Enantioselective C–S Bond Formation

Despite the substantial progress achieved in sulfur chemistry, catalytic enantioselective C–S bond formation remains considerably more challenging than the corresponding C–C, C–N, or C–O bond-forming reactions. One of the major obstacles originates from the intrinsic nucleophilicity and coordinative ability of sulfur-containing substrates. Thiols, sulfides, and related sulfur nucleophiles can strongly coordinate to transition-metal centers, frequently resulting in catalyst poisoning and decreased catalytic activity. Such undesired interactions often complicate catalyst design and limit substrate compatibility [15]. Another challenge arises from the high reactivity of sulfur-based intermediates. Sulfur-centered species can readily undergo oxidation, disproportionation, or side reactions under catalytic conditions. Furthermore, many sulfur-containing compounds exhibit rapid interconversion between stereoisomeric forms, leading to erosion of stereochemical purity. The generation and preservation of stereogenic sulfur centers therefore requires careful control of both kinetic and thermodynamic factors [16,17]. The stereochemical complexity of sulfur compounds introduces additional difficulties. Enantioselective C–S bond-forming reactions may generate chirality at carbon, sulfur, or both centers simultaneously. Consequently, catalyst systems must often control multiple stereochemical elements while suppressing competing pathways. This challenge is particularly pronounced in the synthesis of sulfoximines, sulfilimines, and sulfinamides, which have recently emerged as valuable targets in medicinal chemistry [6,18]. From a mechanistic perspective, the design of enantioselective catalytic systems is further complicated by the diverse reaction pathways available for sulfur reagents. Depending on reaction conditions, sulfur-containing substrates may participate in ionic, radical, organometallic, or photochemical processes. Achieving high levels of enantiocontrol in such mechanistically diverse transformations remains an active area of research [19,20].

Nevertheless, recent developments in chiral ligand design, dual catalysis, photoredox chemistry, and computationally guided catalyst optimization have led to remarkable breakthroughs. These advances have enabled the development of increasingly efficient catalytic systems capable of delivering sulfur-containing molecules with excellent enantioselectivities and broad substrate scope.

### 1.3. Scope and Organization of This Review

Several excellent reviews have summarized general methods for C–S bond construction, sulfur-centered radical chemistry, sulfur(VI) functional groups, and the synthesis of organosulfur compounds [11,12,13,14,21,22,23]. However, a comprehensive analysis specifically focused on catalytic enantioselective C–S bond-forming reactions remains lacking. The present review aims to provide a critical overview of catalytic methodologies in which stereochemical information is introduced during the formation of a carbon–sulfur bond. Particular emphasis is placed on transition-metal-catalyzed, organocatalytic, photocatalytic, and emerging electrocatalytic approaches that enable the asymmetric construction of sulfur-containing molecular architectures. The review is organized according to the nature of the catalytic platform employed. Following an overview of transition-metal-catalyzed transformations, organocatalytic strategies and photochemical methodologies are discussed. Emerging electrochemical approaches are subsequently highlighted. Special attention is devoted to the generation of sulfur-centered stereogenicity, mechanistic aspects of enantioinduction, and applications in pharmaceutical synthesis, natural product construction, and functional materials. By comparing catalytic systems, mechanistic paradigms, and synthetic applications, we aim to identify current limitations and future opportunities in the rapidly evolving field of enantioselective C–S bond formation.

## 2. Classification of Enantioselective C–S Bong Forming Reactions

The catalytic enantioselective construction of carbon–sulfur bonds has evolved into a diverse field encompassing a broad spectrum of mechanistically distinct transformations. Depending on the nature of the sulfur reagent, catalytic platform, and stereodetermining step, asymmetric C–S bond-forming reactions may proceed through ionic, organometallic, radical, or photochemically generated intermediates. Over the past two decades, advances in catalyst design and reaction engineering have significantly expanded the synthetic toolbox available for the preparation of sulfur-containing compounds possessing stereogenic carbon and sulfur centers [12,13,14,17]. Historically, transition-metal-catalyzed allylic substitution reactions constituted some of the earliest and most successful approaches to enantioselective C–S bond formation. Palladium- and iridium-based catalytic systems enabled the synthesis of chiral allylic sulfides with high levels of enantiocontrol while establishing fundamental principles governing sulfur nucleophile compatibility in asymmetric catalysis [24,25]. Subsequent developments in asymmetric conjugate additions, cross-coupling reactions, and C–H functionalization methodologies considerably broadened the scope of accessible sulfur-containing molecular architectures [15,16,18]. In parallel, organocatalytic approaches emerged as powerful alternatives to metal-based systems. Chiral phosphoric acids, cinchona alkaloids, thioureas, squaramides, and *N*-heterocyclic carbenes have been successfully employed in various asymmetric sulfenylation and sulfur-transfer reactions. Such methodologies often provide excellent functional-group tolerance and avoid issues associated with residual metal contamination, making them particularly attractive for applications in medicinal chemistry and pharmaceutical synthesis [26,27,28]. More recently, visible-light photocatalysis and dual catalytic strategies have opened new opportunities for asymmetric sulfur chemistry. The controlled generation of sulfur-centered radicals under mild conditions has enabled previously inaccessible transformations while offering novel pathways for stereocontrol through synergistic interactions between photoredox catalysts and chiral catalytic systems. Furthermore, electrochemical methods have emerged as promising sustainable alternatives, although their application to enantioselective C–S bond formation remains relatively underdeveloped [29,30,31,32]. Based on the dominant catalytic platform and mechanistic characteristics, currently available enantioselective C–S bond-forming methodologies can be classified into the categories summarized in Table 1.

The classification presented in Table 1 highlights the remarkable diversification of asymmetric C–S bond-forming methodologies. Although transition-metal-catalyzed processes continue to dominate the field in terms of substrate scope and synthetic maturity, organocatalytic and photochemical approaches have gained increasing prominence during the last decade. Particularly noteworthy is the growing number of synergistic catalytic systems combining multiple activation modes, including photoredox/transition-metal catalysis and photoredox/organocatalysis, which often provide improved reactivity and stereochemical control [23,33,34]. Another important trend concerns the increasing emphasis on sulfur-centered stereogenicity. Whereas most early studies focused on the generation of chirality at carbon atoms adjacent to sulfur functionalities, contemporary research increasingly targets the direct catalytic synthesis of configurationally stable sulfur-stereogenic compounds such as sulfoximines, sulfilimines, and sulfinamides. These structural motifs have attracted considerable attention owing to their emerging importance in medicinal chemistry, agrochemical research, and asymmetric catalysis [10,11,21,22]. The following sections discuss each catalytic platform in detail, with particular emphasis on catalyst design, mechanistic aspects of stereocontrol, substrate scope, synthetic applications, and future challenges.

## 3. Transition-Metal-Catalyzed Enantioselective C–S Bond Formation

Transition-metal catalysis has played a pivotal role in the development of enantioselective carbon–sulfur bond-forming reactions and remains one of the most powerful approaches for the synthesis of chiral organosulfur compounds. The unique ability of transition metals to activate electrophiles, nucleophiles, and unsaturated substrates under mild reaction conditions has enabled the development of numerous catalytic methodologies for the stereoselective construction of C–S bonds [12,13,14,35]. Among the various catalytic platforms available, transition-metal-catalyzed processes often provide superior levels of reactivity, substrate scope, and stereochemical control. In particular, palladium-, copper-, rhodium-, nickel-, and iridium-based catalytic systems have proven highly effective in a wide range of transformations, including allylic substitution, conjugate addition, cross-coupling, hydrofunctionalization, and C–H functionalization reactions [12,13,14,15]. These methodologies have significantly expanded the accessibility of sulfur-containing compounds possessing stereogenic carbon centers as well as more challenging sulfur-stereogenic architectures. Despite these advances, the development of transition-metal-catalyzed asymmetric C–S bond-forming reactions has been accompanied by several fundamental challenges. Sulfur-containing substrates often exhibit strong coordination to metal centers, which may lead to catalyst deactivation or reduced stereocontrol. Furthermore, the high nucleophilicity and redox activity of sulfur reagents can promote competing pathways, thereby complicating catalyst design and reaction optimization [36,37]. Consequently, the identification of suitable chiral ligand frameworks capable of balancing reactivity and selectivity has become a central theme in this field. Over the past two decades, substantial progress in ligand engineering, mechanistic understanding, and catalyst development has transformed transition-metal-catalyzed C–S bond formation into a mature and versatile area of asymmetric synthesis. Particularly noteworthy are recent advances in copper- and nickel-catalyzed transformations, which have enabled access to previously challenging substrate classes while improving sustainability and atom economy [16,18]. The following sections summarize the major transition-metal-catalyzed strategies for enantioselective C–S bond formation, focusing on catalyst design, mechanistic aspects of stereocontrol, synthetic scope, and practical applications.

An alternative approach to asymmetric C–S bond formation was demonstrated by Fasan and co-workers using engineered myoglobin-based catalysts. These biocatalysts promoted intermolecular carbene S–H insertion with a broad range of thiols and α-diazoester-derived carbene precursors, providing thioethers in high conversions. Rational modification of the protein active site enabled asymmetric induction, with selected myoglobin variants providing up to 49% *ee* [38].

### 3.1. Palladium-Catalyzed Reactions

Palladium-catalyzed asymmetric carbon–sulfur bond-forming reactions represent one of the earliest and most extensively studied classes of enantioselective transformations in organosulfur chemistry. The success of palladium catalysis largely originates from its unique ability to generate well-defined π-allyl intermediates that can undergo highly stereoselective nucleophilic substitution in the presence of suitable chiral ligands. Consequently, palladium-catalyzed allylic substitution has become a cornerstone methodology for the synthesis of chiral sulfur-containing compounds and has significantly influenced the development of modern asymmetric catalysis [35,39,40]. The pioneering studies of Trost, Hayashi, Helmchen, and co-workers established the fundamental principles governing palladium-catalyzed asymmetric allylic alkylation reactions and demonstrated that sulfur nucleophiles could participate in such processes with excellent regio- and enantioselectivity [24,25,35,41,42,43,44,45,46,47,48,49,50,51,52,53,54,55,56,57,58,59,60,61,62,63]. These early contributions revealed that the stereochemical outcome strongly depends on the structure of both the chiral ligand and the sulfur nucleophile, highlighting the importance of catalyst–substrate interactions during nucleophilic attack on the η^3^-allylpalladium intermediate. Compared with other transition-metal-catalyzed approaches, palladium-catalyzed reactions offer several important advantages, including broad substrate scope, mild reaction conditions, and high functional-group tolerance. Furthermore, the extensive availability of chiral phosphine and phosphoramidite ligands has enabled precise control over stereochemical induction in a variety of C–S bond-forming transformations [42,43]. As a result, palladium catalysis remains one of the most reliable strategies for the preparation of enantioenriched allylic sulfides and related sulfur-containing building blocks. Despite these advantages, several challenges remain. Sulfur nucleophiles often exhibit strong affinity toward palladium centers, potentially leading to catalyst deactivation and reduced catalytic efficiency. Moreover, achieving high enantioselectivity can be particularly demanding when less reactive or sterically hindered sulfur nucleophiles are employed [36]. Nevertheless, continuous advances in ligand design and mechanistic understanding have led to increasingly efficient catalytic systems and have expanded the synthetic utility of palladium-catalyzed asymmetric C–S bond formation. The following sections summarize the major developments in palladium-catalyzed enantioselective C–S bond-forming reactions, with particular emphasis on allylic substitution processes, catalyst design, stereochemical models, and synthetic applications.

**Scheme 1:**
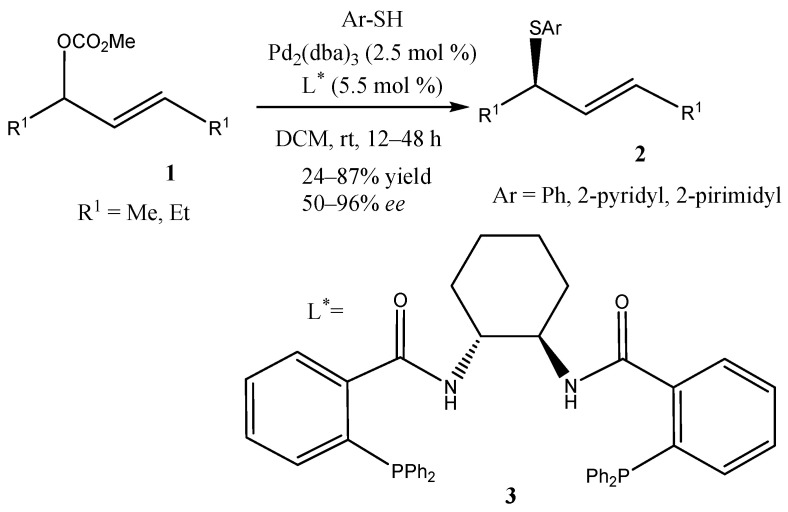
Asymmetric synthesis of allylic sulfides.

Among all palladium-catalyzed enantioselective C–S bond-forming processes, asymmetric allylic substitution remains by far the most extensively investigated and synthetically useful transformation. The reaction proceeds through the generation of a chiral η^3^-allylpalladium intermediate, followed by stereoselective attack of a sulfur nucleophile. Owing to the well-established mechanistic framework of the Tsuji–Trost reaction, allylic sulfides can often be obtained with excellent regioselectivity and high levels of enantiocontrol. Recent developments have focused on expanding the range of sulfur nucleophiles, improving catalyst robustness toward sulfur poisoning, and broadening substrate scope. One of the landmark studies demonstrating the viability of sulfur nucleophiles in Pd-catalyzed AAA was reported by Gais and co-workers [44]. Using Pd_2_(dba)_3_ in combination with Trost-type ligands, various thiophenols and heteroaryl thiols were reacted with racemic allylic carbonates **1** to furnish allylic sulfides **2** in good yields and up to 96% *ee* (Scheme 1). This work clearly established that sulfur nucleophiles could participate efficiently in asymmetric allylic substitution without complete catalyst deactivation.

**Scheme 2:**
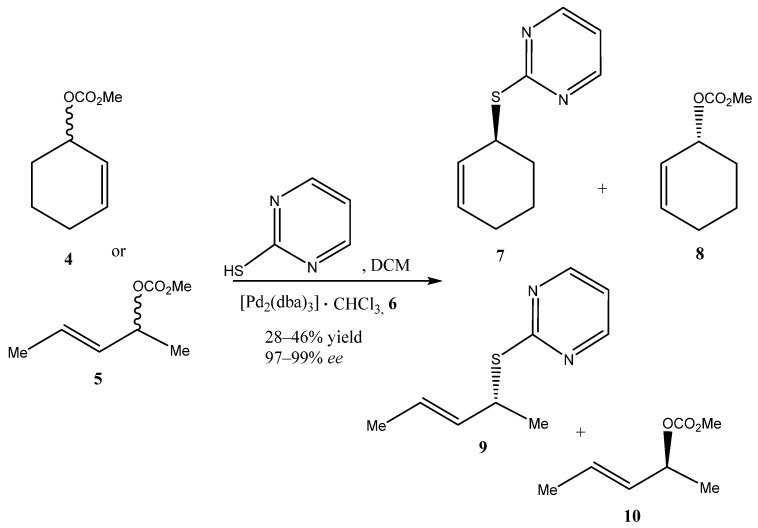
Palladium–catalyzed kinetic resolution of cyclic and acyclic carbonates.

In a subsequent study, Gais and co-workers extended the methodology to the kinetic resolution of racemic allylic carbonates **4**, **5** using sulfur nucleophiles such as sulfinates and heterocyclic thiols [45]. The process delivered allylic sulfides and sulfones with excellent selectivities and demonstrated the remarkable stereochemical control achievable with carefully designed bisphosphine ligands (Scheme 2).

**Scheme 3:**
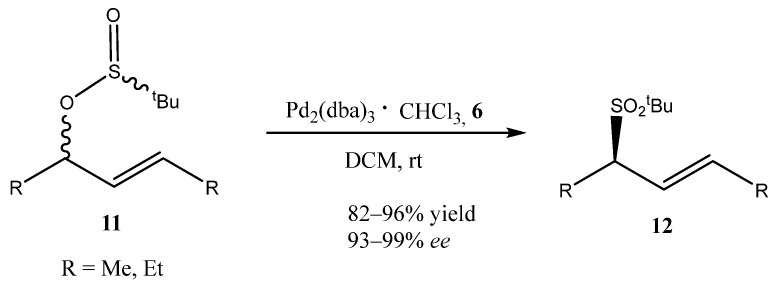
Pd–catalyzed enantioselective 1,3–rearrangement of allylic sulfinates.

In a significant extension of their earlier studies, Gais et al. reported a highly enantioselective palladium-catalyzed 1,3-rearrangement of racemic allylic sulfinates **11**, providing direct access to enantioenriched allylic sulfones [46]. Using Pd_2_(dba)_3_·CHCl_3_ **6** in combination with the chiral bisphosphine ligand BPA, both cyclic and acyclic allylic sulfinates were efficiently converted into the corresponding allylic sulfones **12** in high yields (82–96%) and excellent enantioselectivities (93–99% *ee*). Mechanistic investigations suggested that the transformation proceeds through the generation of a cationic π-allylpalladium intermediate and a sulfinate anion, followed by stereoselective recombination. Furthermore, the authors demonstrated that the rearrangement could be accompanied by highly efficient kinetic resolution of the starting allylic sulfinate, enabling simultaneous access to enantioenriched substrates and products. This work represented one of the earliest examples of exploiting Pd–catalyzed allylic rearrangements for the asymmetric synthesis of sulfur-containing compounds and highlighted the synthetic potential of allylic sulfinate–sulfone interconversions (Scheme 3).

**Scheme 4:**
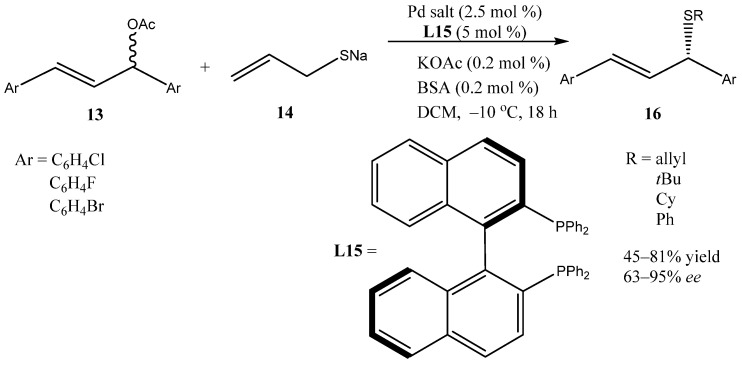
Pd–catalyzed synthesis of thioethers.

In 2017, Zhao and co-workers reported a palladium-catalyzed (**L15**) asymmetric allylic thioetherification of racemic allylic carbonates **13** using sodium sulfinates **14** as sulfur nucleophiles [47]. The protocol furnished a variety of chiral allylic thioethers **16** in high yields and excellent enantioselectivities (up to 97% *ee*), demonstrating the synthetic utility of sulfinate nucleophiles in Pd-catalyzed asymmetric allylic substitution. The reaction proceeded through a π-allylpalladium intermediate and significantly expanded the scope of enantioselective C–S bond-forming processes based on allylic substitution chemistry (Scheme 4).

### 3.2. Copper-Catalyzed Reactions

Copper catalysis has emerged as one of the most versatile and sustainable approaches for enantioselective carbon–sulfur bond formation. Compared with palladium-based systems, copper catalysts offer several practical advantages, including lower cost, reduced toxicity, and the ability to operate through diverse mechanistic pathways involving ionic, radical, and organometallic intermediates. As a result, copper-catalyzed methodologies have significantly expanded the synthetic toolbox available for the preparation of enantioenriched sulfur-containing compounds [48,49]. Particularly noteworthy is the remarkable compatibility of copper catalysts with a wide range of sulfur nucleophiles, including thiols, thiolates, sulfinates, and sulfur-centered radical precursors. The combination of copper salts with chiral bisoxazoline, phosphine, *N*-heterocyclic carbene, and related ligand systems has enabled highly enantioselective transformations leading to allylic sulfides, β-thio carbonyl compounds, sulfur-containing heterocycles, and other valuable organosulfur architectures [50,51,52]. In contrast to palladium-catalyzed allylic substitution reactions, which historically dominated the field, copper catalysis has facilitated the development of fundamentally different reaction classes. These include asymmetric conjugate additions, propargylic substitutions, radical difunctionalizations of alkenes, and photochemically assisted processes. Such transformations have broadened both the structural diversity of accessible products and the mechanistic landscape of enantioselective C–S bond formation [53]. Recent years have witnessed particularly rapid growth in copper-catalyzed sulfur chemistry, driven by advances in ligand design, photoredox catalysis, and radical-mediated reaction development. These innovations have enabled the efficient construction of stereogenic carbon centers adjacent to sulfur atoms under increasingly mild and environmentally benign conditions, further establishing copper as a key metal in modern asymmetric organosulfur synthesis.

A major advance in this area was reported by Liu and co-workers, who developed a general copper-catalyzed enantioconvergent C(sp^3^)–S cross-coupling based on a biomimetic radical homolytic substitution strategy [54]. The method enabled the conversion of racemic alkyl electrophiles into enantioenriched sulfur-containing products through radical intermediates and chiral copper catalysis, providing a powerful platform for asymmetric C–S bond construction. An early example of catalytic asymmetric C–S bond formation was reported by Zhou and co-workers, who developed a copper-catalyzed enantioselective insertion of metal carbenoids into S–H bonds. Using a chiral spirobisoxazoline–copper complex, a range of α-mercapto esters were obtained in high yields and with moderate to good enantioselectivities, reaching up to 85% *ee* [55].

**Scheme 5:**
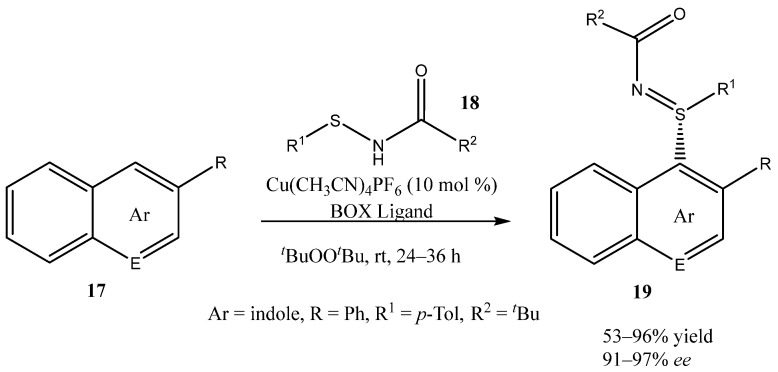
Copper–mediated asymmetric sulfilimination of aromatic compounds.

In 2025, Li and co-workers reported a copper-catalyzed asymmetric C–H sulfilimination of electron–rich (hetero)arenes **17** through a HAT-primed C–S radical–radical coupling strategy [56]. The method enabled the direct conversion of indoles and naphthols into chiral sulfilimines **19** via oxidative coupling with sulfenamides **18** under mild reaction conditions, employing either molecular oxygen from air or organic peroxides as terminal oxidants (Scheme 5). A broad range of substrates was accommodated, affording the corresponding sulfilimines **19** with excellent regioselectivities and high levels of enantiocontrol despite the inherent configurational lability of sulfur-stereogenic sulfilimine products. Mechanistic investigations, supported by DFT calculations, revealed an unusual dual hydrogen atom transfer (HAT) pathway. The key C–S bond-forming event was proposed to proceed through an outer-sphere radical–radical coupling process involving concerted arene hydrogen abstraction and C–S bond formation within an eight-membered transition state.

**Scheme 6:**
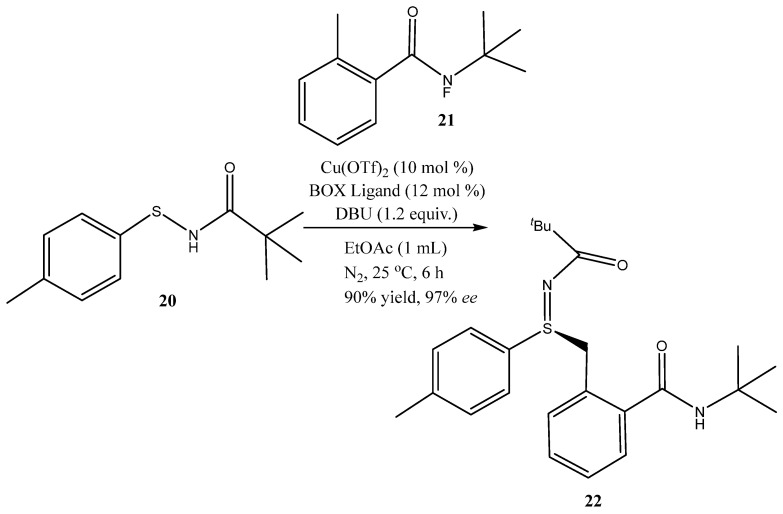
Copper–catalyzed asymmetric sulfilimination.

In 2026, the same group developed a copper-catalyzed asymmetric sulfilimination of diverse radical precursors through a nitrogen-centered radical relay strategy, providing direct access to sulfur-stereogenic sulfilimines **22** [57]. The method employs readily available radical precursors **21** and sulfenamides **20** under mild catalytic conditions, enabling the efficient construction of S(IV)-stereogenic sulfur centers with high levels of enantioselectivity (Scheme 6). A broad substrate scope was demonstrated, including benzylic, allylic, and alkyl radical precursors, affording structurally diverse sulfilimines **22** in good to excellent yields and enantiomeric ratios. Mechanistic studies suggested the involvement of nitrogen-centered radical intermediates generated under copper catalysis, which subsequently engage in enantioselective C–S bond-forming processes.

**Scheme 7:**
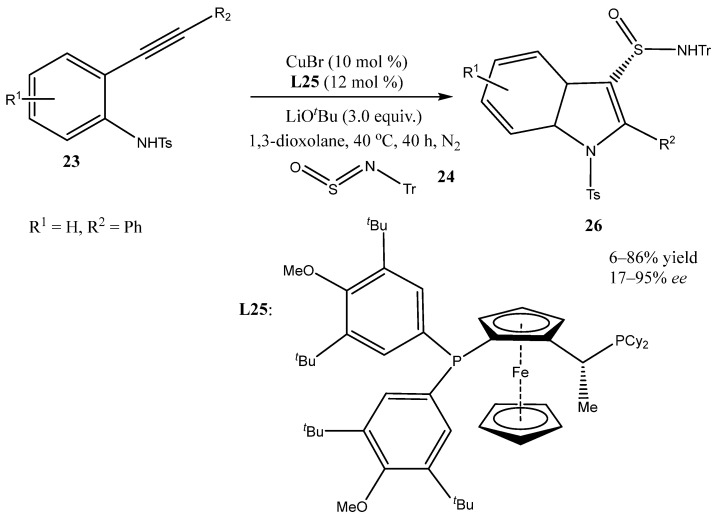
Copper–catalyzed asymmetric cyclizative sulfinamidation.

In 2025, Shi et al. reported a copper-catalyzed asymmetric cyclizative sulfinamidation of *ortho*-alkynylanilines **23** with sulfinylamines **24**, providing efficient access to indole derivatives bearing sulfur-stereogenic sulfinamide motifs **26** [58]. The reaction proceeds through a cascade process involving intramolecular cyclization and simultaneous C–S bond formation, enabling the construction of both S(IV)-stereogenic centers and, in many cases, atropisomeric axial chirality within a single transformation (Scheme 7). A broad range of substrates was tolerated, affording the desired products in good yields and with excellent enantioselectivities in the presence of ligand **L25**. Mechanistic studies, supported by DFT calculations, revealed the origin of stereocontrol and highlighted the crucial role of the chiral copper catalyst in governing both sulfur-centered and axial chirality.

**Scheme 8:**
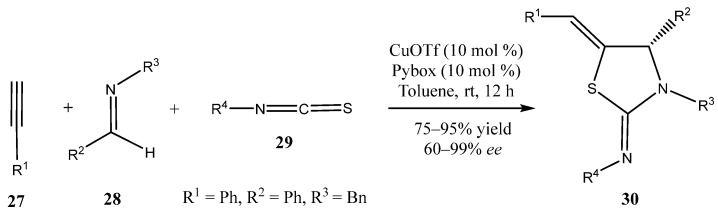
Asymmetric synthesis of thiazolidine-2-imines.

Ranjan and co-workers reported a copper-catalyzed asymmetric three-component alkynylation/hydrothiolation cascade involving terminal alkynes **27**, imines **28**, and isothiocyanates **29**. Using a chiral Cu(I)/pybox catalyst system, the authors developed an efficient one-pot protocol for the synthesis of enantioenriched thiazolidine-2-imines **30** in good to excellent yields and with outstanding enantioselectivities of up to 99% *ee* (Scheme 8) [59]. The transformation proceeds through an initial enantioselective alkynylation of the imine, followed by addition of the resulting propargylamine to an isothiocyanate and a highly regioselective intramolecular 5-*exo*-*dig* hydrothiolation, which constitutes the key C–S bond-forming step. This methodology provides a concise route to sulfur-containing heterocycles and demonstrates the utility of copper catalysis in asymmetric cascade processes.

Examples of copper-catalyzed asymmetric C–S bond formation remain sparsely represented in the literature. The earlier review article only shows classical transformations leading to this type of bond formation without stereochemical control [60].

### 3.3. Rhodium-Catalyzed Processes

Rhodium catalysis has emerged as a powerful platform for enantioselective bond-forming reactions owing to the unique reactivity of Rh(I) and Rh(III) complexes and the availability of highly efficient chiral ligand systems. In the context of C–S bond formation, rhodium catalysts have enabled several asymmetric transformations, including enantioselective carbenoid insertions [61], and hydrothiolations [62]. Compared with palladium- and copper-catalyzed methodologies, rhodium-catalyzed processes often exhibit complementary substrate scope and distinct mechanistic pathways, allowing access to structurally diverse sulfur-containing compounds with high levels of stereocontrol. In particular, the strong ability of rhodium complexes to activate unsaturated substrates and to promote stereoselective migratory insertion processes has been successfully exploited in the synthesis of chiral sulfides, sulfoxides, and sulfur-containing heterocycles. Recent developments have further demonstrated the potential of rhodium catalysis in the construction of sulfur-stereogenic molecules and other architecturally complex sulfur-containing frameworks.

**Scheme 9:**
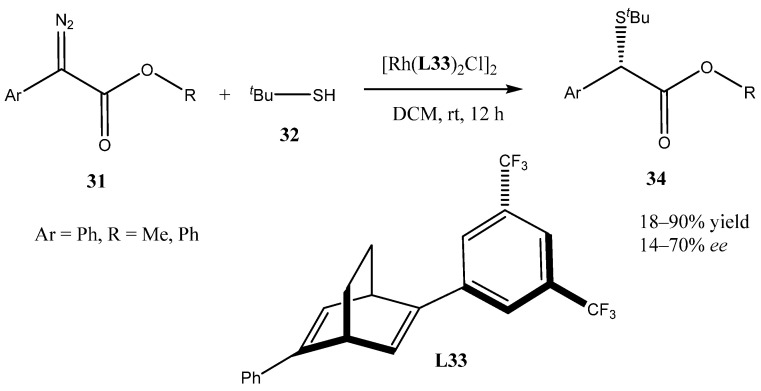
Rhodium(I)–catalyzed asymmetric carbene insertion into the S–H bond.

In 2022, Xu et al. reported the first Rh(I)-catalyzed asymmetric carbenoid S–H insertion for the construction of C–S bonds [61]. Using a chiral diene–rhodium catalyst, α-diazoarylacetates **31** underwent highly efficient insertion into the S–H bond of *tert*-butyl mercaptan **32** under mild reaction conditions, providing a range of α-thioesters **34** bearing sulfur-substituted stereogenic centers (Scheme 9). The methodology afforded the desired products in high yields and moderate to good enantioselectivities, demonstrating that chiral Rh(I) complexes (**L33**) can effectively control stereochemistry during intermolecular carbene insertion into S–H bonds. Xu and co-workers developed a highly enantioselective S–H bond insertion through cooperative catalysis by a dirhodium(II) complex and a chiral spiro phosphoric acid [62]. The reaction provided α-mercapto esters in high yields and with excellent enantioselectivities of up to 98% *ee*, demonstrating the effectiveness of cooperative metal/organocatalytic strategies for asymmetric C–S bond formation.

**Scheme 10:**
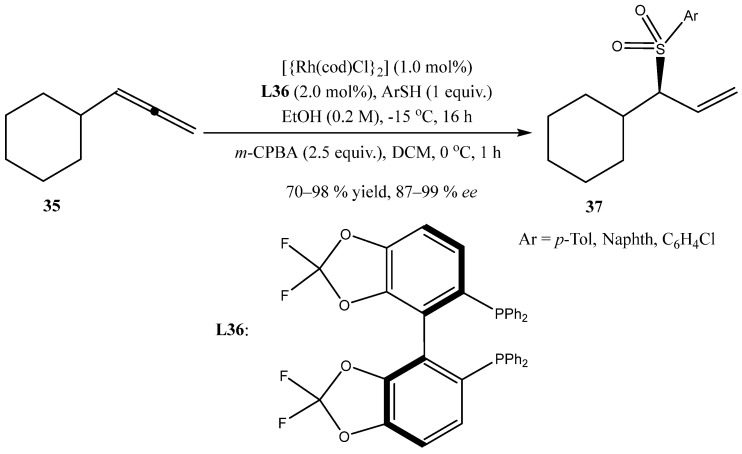
Rh–promoted asymmetric addition of thiols to allenes.

Pritzius and Breit developed a highly regio- and enantioselective Rh-catalyzed intermolecular hydrothiolation of terminal allenes **35** with aromatic and functionalized aliphatic thiols (Scheme 10) [63]. By employing two complementary chiral rhodium catalyst systems **L36**, the reaction provided branched allylic thioethers **37** with high regio- and enantioselectivities and broad functional-group tolerance. The atom-economic nature of the transformation enables direct C–S bond formation without prefunctionalization of the allene substrate. Moreover, oxidation of the resulting thioethers furnished the corresponding allylic sulfones in essentially enantiomerically pure form. Labeling experiments provided initial mechanistic insight into this rhodium-catalyzed asymmetric hydrothiolation.

Some examples of rhodium-assisted asymmetric hydrofunctionalization of alkenes were reviewed in 2026 [64]. Although numerous rhodium-catalyzed C–S bond-forming reactions have been developed through C–H activation strategies [65,66], asymmetric variants still remain comparatively scarce.

### 3.4. Nickel-Catalyzed Reactions

**Scheme 11:**
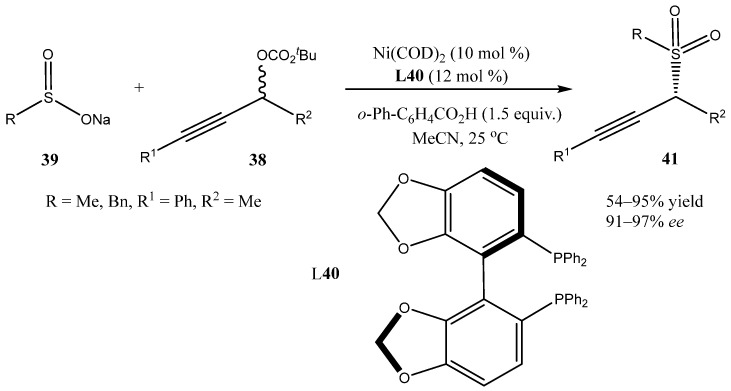
Ni–catalyzed asymmetric propargylic sulfonation.

Nickel catalysis has emerged as a powerful alternative to palladium and copper catalysis in carbon–heteroatom bond-forming reactions owing to the unique electronic properties, earth abundance, and low cost of nickel complexes. In the field of C–S bond formation, nickel catalysts have demonstrated remarkable versatility in cross-coupling, reductive coupling, and radical-mediated transformations, enabling the efficient synthesis of a wide range of sulfur-containing compounds. In particular, the ability of nickel to engage in both two-electron and single-electron pathways has opened new opportunities for the development of stereoselective and enantioselective sulfur-functionalization reactions [66,67,68,69]. Compared with palladium- and rhodium-catalyzed processes, nickel-catalyzed methodologies often exhibit complementary reactivity profiles and broader compatibility with unconventional electrophiles and radical precursors. Recent advances in ligand design and mechanistic understanding have further facilitated the development of asymmetric nickel-catalyzed C–S bond-forming reactions, including cross-coupling processes and radical relay transformations leading to enantioenriched sulfur-containing molecules. Although this area remains less developed than other branches of asymmetric nickel catalysis, recent achievements clearly demonstrate the growing potential of nickel complexes in the construction of chiral organosulfur compounds [70,71]. In 2026, Guo and co-workers reported a nickel-catalyzed asymmetric sulfonation of racemic propargylic carbonates **38** using sodium sulfinates **39** as sulfur nucleophiles [72]. Through a highly regio- and enantioselective propargylic substitution process, a broad range of propargylated, allenylated, and 1,3-dienyl sulfones **41** were obtained in good yields and excellent enantioselectivities (Scheme 11). The methodology relies on chiral nickel catalysis (**L40**) to control both regioselectivity and stereochemistry during C–S bond formation and provides direct access to valuable enantioenriched sulfone building blocks. Furthermore, the synthetic utility of the products was demonstrated through their transformation into diverse sulfur-containing molecules, highlighting the potential of nickel catalysis for asymmetric C–S bond construction.

**Scheme 12:**
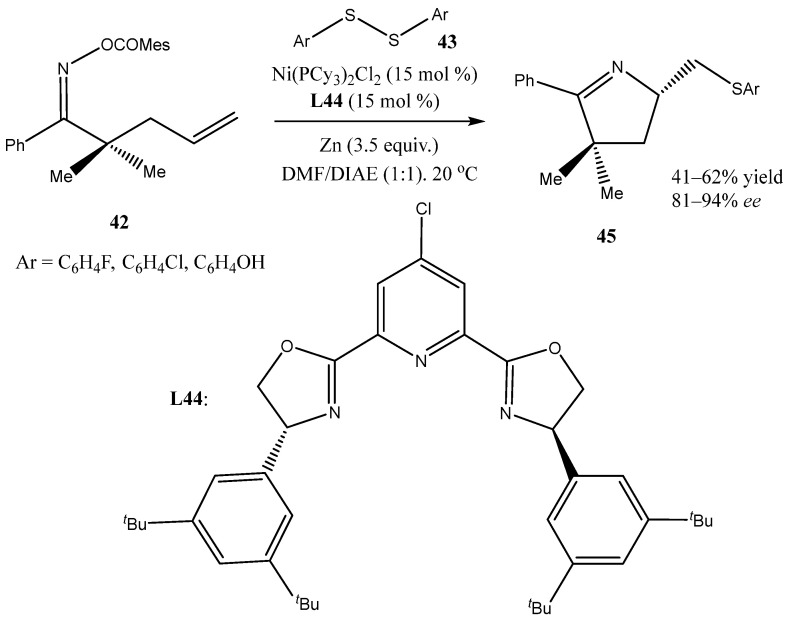
Ni–catalyzed enantioselective reductive *N*-cyclization-thiolation reaction.

In 2024, Shu and co-workers reported a nickel-catalyzed enantioselective reductive *N*-cyclization–thiolation of alkene-tethered oxime esters **42** with disulfides **43** [73]. The transformation proceeds in the presence of chiral ligand **L44** through a reductive aza-Heck cyclization followed by enantioselective C–S bond formation, providing sulfide-containing pyrrolines **45** bearing stereogenic centers in high enantiomeric purity (Scheme 12). The method operates under mild conditions and exhibits broad functional-group tolerance, accommodating substrates containing alkynes, phenols, anilines, amides, nitriles, and aryl bromides. This constitutes an elegant example of asymmetric nickel catalysis that combines heterocycle construction and C–S bond formation in a single catalytic sequence.

**Scheme 13:**
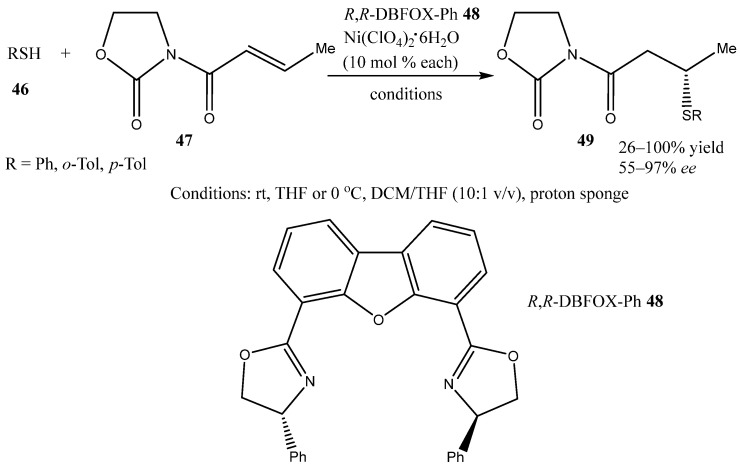
Ni–promoted asymmetric addition of thiols to oxazolidinones.

Kanemasa and co-workers reported one of the earliest examples of nickel-catalyzed enantioselective C–S bond formation through the conjugate addition of thiols **46** to α,β-unsaturated *N*-acyl oxazolidinones **47** [74]. Using a chiral Ni(II)/DBFOX-Ph **48** catalyst system, a variety of thiols were added to 3-(2-alkenoyl)-2-oxazolidinones to afford β-sulfur-substituted products **49** in high yields and excellent enantioselectivities (Scheme 13). The reaction proceeds via asymmetric Michael addition, generating a new C–S bond and a stereogenic carbon center in a single step. This study demonstrated the potential of chiral nickel complexes for controlling stereochemistry in sulfur-functionalization reactions and represents a landmark contribution to asymmetric C–S bond formation.

**Scheme 14:**
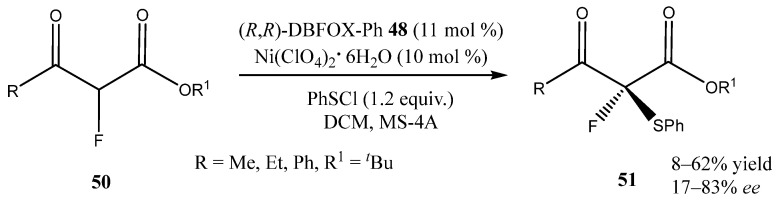
Ni–promoted asymmetric synthesis of α-fluoro-α-sulfenyl-β-ketoesters.

Ishimaru and co-workers described an enantioselective α-sulfenylation of fluorinated β-ketoesters **50** catalyzed by a chiral Ni(II)/DBFOX-Ph **48** complex [75]. The reaction enabled the formation of C–S bonds at the α-position of β-ketoesters, affording α-fluoro-α-sulfenylated products **51** bearing quaternary stereogenic centers in good yields and high enantioselectivities (Scheme 14).

### 3.5. Iridium-Catalyzed Reactions

Iridium-catalyzed asymmetric transformations have become an important component of modern synthetic chemistry owing to their exceptional ability to control regio-, diastereo-, and enantioselectivity in carbon–carbon and carbon–heteroatom bond-forming reactions. In particular, chiral iridium complexes have proven highly effective in asymmetric allylic substitution reactions, often displaying complementary regioselectivity to palladium catalysts and enabling the efficient construction of stereogenic centers under mild reaction conditions [25,76,77]. In the context of organosulfur chemistry, iridium catalysis has provided access to a variety of enantioenriched sulfides and sulfur-containing heterocycles through asymmetric allylic substitution and related processes involving sulfur nucleophiles. The combination of highly tunable chiral phosphoramidite and phosphoramidite–olefin ligands with the unique reactivity of π-allyliridium intermediates has enabled the development of efficient methodologies for enantioselective C–S bond formation. Although fewer examples have been reported compared with palladium-catalyzed systems, iridium-catalyzed processes frequently deliver excellent regio- and enantioselectivities and constitute a valuable complement to other transition-metal-catalyzed approaches [78,79].

**Scheme 15:**
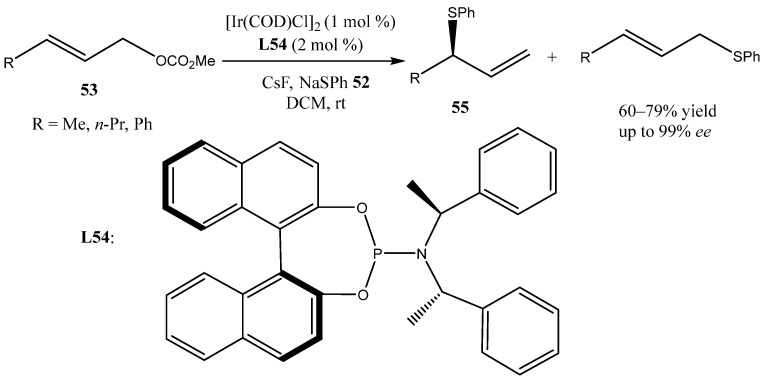
Ir–catalyzed allylation of NaSPh.

Zhao et al. reported a regio- and enantioselective iridium-catalyzed allylic substitution between thiophenol **52** and allylic carbonates **53**, providing direct access to enantioenriched allyl phenyl sulfides **55** (Scheme 15) [80]. Using a chiral iridium catalyst **L54**, the reaction proceeded with excellent branched-to-linear selectivity and high enantiocontrol, affording a variety of allylic sulfides **55** in high yields and up to excellent enantiomeric excesses.

**Scheme 16:**
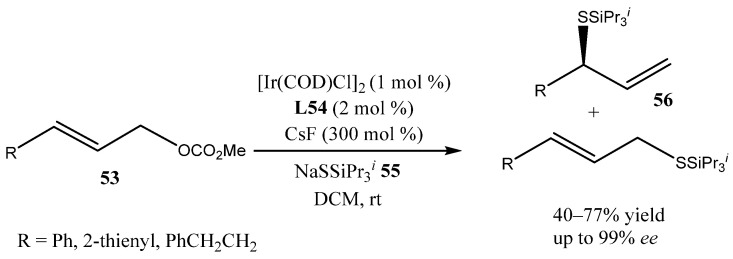
Ir–promoted asymmetric allylation of allyl methyl carbonates.

Building upon their earlier studies on aromatic thiols, the same group developed an iridium-catalyzed asymmetric allylation of aliphatic thiols with allylic carbonates using the same catalytic system [81]. The method provided a straightforward route to branched allylic sulfides through highly regioselective allylic substitution, furnishing the products in good yields and excellent enantioselectivities. Notably, the protocol tolerated a variety of primary and secondary aliphatic thiols, demonstrating the broad applicability of iridium-catalyzed asymmetric C–S bond formation. Zhao et al. also expanded the scope of iridium-catalyzed asymmetric allylic substitution by employing sodium triisopropylsilanethiolate as a masked thiol equivalent [82]. The reaction of allylic carbonates **53** with TIPS-protected sulfur nucleophiles **55** afforded branched allylic sulfides **56** in moderate to good yields, excellent regioselectivities, and up to 89% *ee* (Scheme 16). Importantly, the resulting silyl-protected sulfides could be readily converted into optically enriched thiols, providing a practical strategy for the synthesis of chiral sulfur-containing building blocks.

**Scheme 17:**
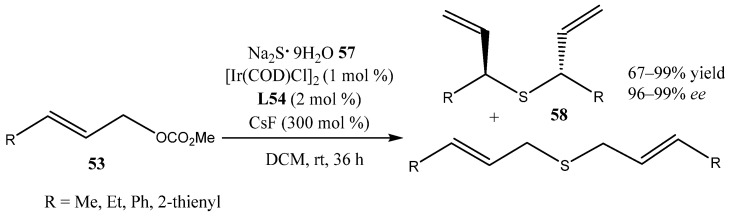
Ir–catalyzed double allylation of sodium sulfide with allyl methyl carbonates.

In the same year, Zhao and co-workers reported a one-pot iridium-catalyzed asymmetric double allylation of sodium sulfide, providing an efficient route to C_2_-symmetric bis(allylic) sulfides [83]. In this strategy, sodium sulfide **57** acts as a bisnucleophilic sulfur source and undergoes two consecutive enantioselective allylic substitution reactions with allylic carbonates **53** under the control of a chiral iridium catalyst **L54** (Scheme 17). The resulting products **58** were obtained in high yields, excellent enantioselectivities (up to 99% *ee*), and high diastereoselectivities, generating two stereogenic carbon centers through the formation of two new C–S bonds.

**Scheme 18:**
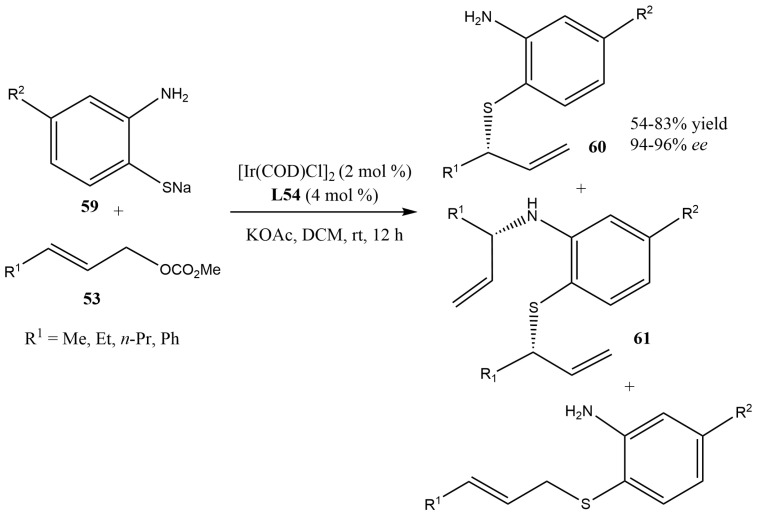
Ir–catalyzed enantioselective allylic alkylation of sodium 2-aminobenzenethiolate.

In 2012, Zhao et al. described an iridium-catalyzed enantioselective allylic substitution of sodium 2-aminobenzenethiolate **59** with a broad range of allylic carbonates **53** [84]. Despite the ambident nature of the nucleophile, the reaction proceeded predominantly through sulfur attack, affording branched allylic sulfides **60** and **61** with excellent regioselectivities and enantioselectivities (up to 97% *ee*) (Scheme 18). The resulting N,S-functionalized intermediates were further transformed into benzo-fused N,S-heterocycles, demonstrating the synthetic utility of the methodology.

**Scheme 19:**
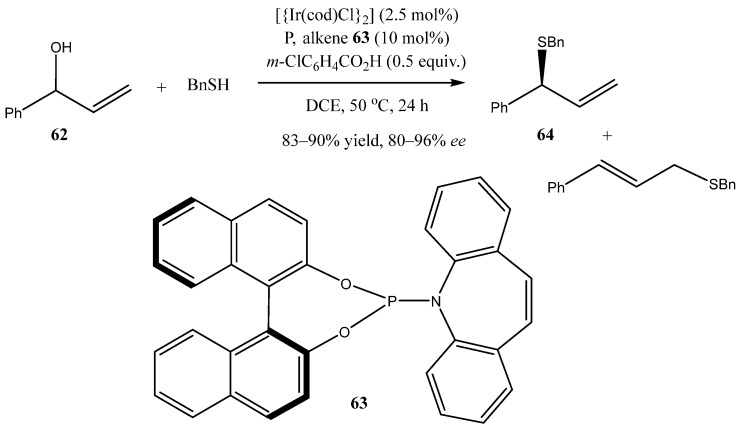
Iridium promoted enantioselective thioetherification.

Finally, in 2012, Roggen and Carreira subsequently developed an enantioconvergent iridium-catalyzed allylic thioetherification of racemic allylic alcohols **62** (Scheme 19) [85]. The combination of an Ir–P,alkene complex **63** and dibutyl phosphate enabled the conversion of both substrate enantiomers into the same enantiomer of the allylic thioether **64**, demonstrating an alternative strategy for asymmetric C–S bond formation based on enantioconvergent catalysis.

## 4. Organocatalytic Enantioselective C–S Bond Formation

Organocatalysis has become one of the most powerful strategies for asymmetric synthesis, providing efficient and environmentally benign alternatives to transition-metal catalysis. Since the pioneering discoveries of proline-catalyzed aldol reactions and hydrogen-bond donor catalysis, a broad range of chiral small organic molecules have been developed as highly effective catalysts for stereoselective bond-forming processes. Owing to their operational simplicity, low toxicity, and often excellent stereochemical control, organocatalytic methods have found widespread application in the synthesis of enantioenriched sulfur-containing compounds [86,87,88,89]. In the context of C–S bond formation, organocatalysis offers several distinct activation modes, including enamine catalysis, iminium catalysis, Brønsted acid and base catalysis, phase-transfer catalysis, hydrogen-bond donor catalysis, and bifunctional activation. These approaches have enabled numerous asymmetric transformations involving sulfur nucleophiles or electrophilic sulfur-transfer reagents, leading to a diverse array of chiral sulfides, thioethers, sulfoxides, sulfoximines, and sulfur-containing heterocycles [26,90,91]. Particularly significant progress has been achieved in asymmetric Michael additions of thiols to activated alkenes, electrophilic sulfenylation reactions, and cascade processes in which C–S bond formation is coupled with the simultaneous generation of one or more stereogenic centers. Recent developments have further expanded the scope of organocatalytic sulfur-functionalization reactions toward increasingly complex molecular architectures and previously inaccessible sulfur-stereogenic compounds. Consequently, organocatalytic enantioselective C–S bond-forming reactions now represent an important and rapidly evolving branch of asymmetric organosulfur chemistry [92,93,94].

### 4.1. Organocatalytic Asymmetric Sulfa-Michael Additions

Among the various organocatalytic strategies for enantioselective C–S bond formation, asymmetric Michael addition of thiols to electron-deficient alkenes represents one of the most extensively investigated and synthetically useful approaches. This transformation provides a direct and atom-economical route to enantioenriched sulfides through the simultaneous formation of a new carbon–sulfur bond and one or more stereogenic centers. Owing to the high nucleophilicity of sulfur-centered nucleophiles and the broad availability of activated Michael acceptors, thiol conjugate additions have become a versatile platform for the synthesis of structurally diverse sulfur-containing compounds [92,95]. The development of organocatalytic asymmetric variants has been driven by the emergence of highly efficient activation modes, including enamine and iminium catalysis, hydrogen-bond donor catalysis, phase-transfer catalysis, and bifunctional organocatalysis. In particular, chiral thioureas, squaramides, cinchona alkaloid derivatives, and primary or secondary amine catalysts have enabled excellent levels of stereocontrol in conjugate additions of both aromatic and aliphatic thiols to a wide range of activated olefins [96,97,98,99,100]. Beyond their fundamental mechanistic interest, organocatalytic asymmetric thio-Michael reactions have found broad application in the synthesis of biologically active molecules, pharmaceutical intermediates, and sulfur-containing building blocks. Furthermore, the mild reaction conditions and metal-free nature of these processes make them attractive alternatives to transition-metal-catalyzed methodologies. As a result, asymmetric thio-Michael additions have become a cornerstone of organocatalytic enantioselective C–S bond-forming chemistry and continue to inspire the development of increasingly efficient catalytic systems.

**Scheme 20:**
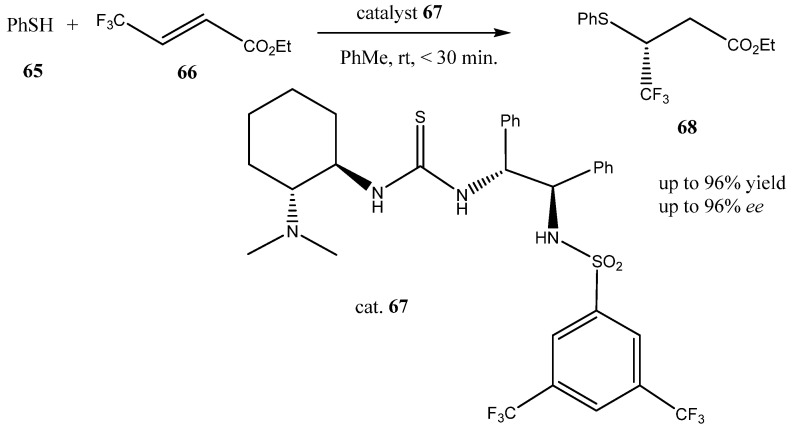
Sulfa–Michael addition of thiofenol to 4,4,4-trifluorocrotonate.

Wang et al. reported an organocatalytic asymmetric sulfa-Michael addition of thiols **65** to 4,4,4-trifluorocrotonates **66** using a cinchona alkaloid-derived bifunctional catalyst **67** [96]. The reaction provided direct access to chiral β-trifluoromethyl sulfides **68** through enantioselective C–S bond formation between aromatic or aliphatic thiols and fluorinated α,β-unsaturated esters (Scheme 20). A broad substrate scope was demonstrated, affording the corresponding thioethers in high yields and good to excellent enantioselectivities. This study highlighted the potential of organocatalytic thio-Michael reactions for the synthesis of fluorinated sulfur-containing building blocks of synthetic and pharmaceutical relevance.

**Scheme 21:**
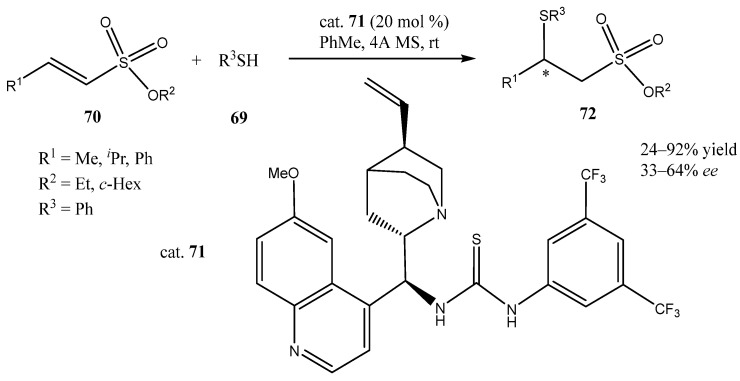
Sulfa–Michael addition of thiols to α,β-unsaturated sulfones.

Enders and Hoffmann reported an organocatalytic asymmetric sulfa-Michael addition of thiols **69** to α,β-unsaturated sulfonates **70** [97]. Using a cinchona alkaloid-derived organocatalyst **71**, a variety of aromatic and aliphatic thiols were efficiently added to activated sulfonate acceptors, furnishing β-sulfonyl-substituted sulfides **72** in good yields and high enantioselectivities (Scheme 21). The methodology expanded the scope of asymmetric thio–Michael reactions beyond traditional Michael acceptors and provided access to synthetically versatile sulfur-containing building blocks.

**Scheme 22:**
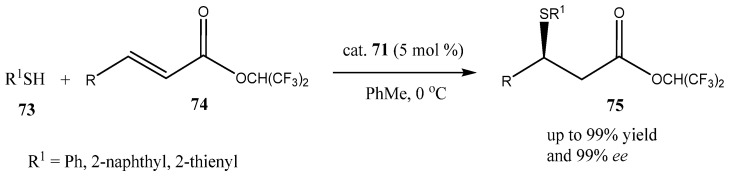
Asymmetric sulfa–Michael addition of thiols to α,β-unsaturated hexafluoroisopropyl esters.

Wang et al. developed a highly efficient organocatalytic asymmetric sulfa-Michael addition of thiols **73** to α,β-unsaturated hexafluoroisopropyl esters **74** [98]. The use of the strongly electron-withdrawing hexafluoroisopropyl ester group significantly enhanced the electrophilicity of the Michael acceptors, enabling highly enantioselective C–S bond formation under mild conditions (Scheme 22). A broad range of thiols and unsaturated esters were successfully employed, affording the corresponding fluorinated sulfides **75** in high yields and excellent enantioselectivities. The synthetic utility of the methodology was further demonstrated through a one-pot synthesis of the biologically active compound (*R*)-thiazesim.

**Scheme 23:**
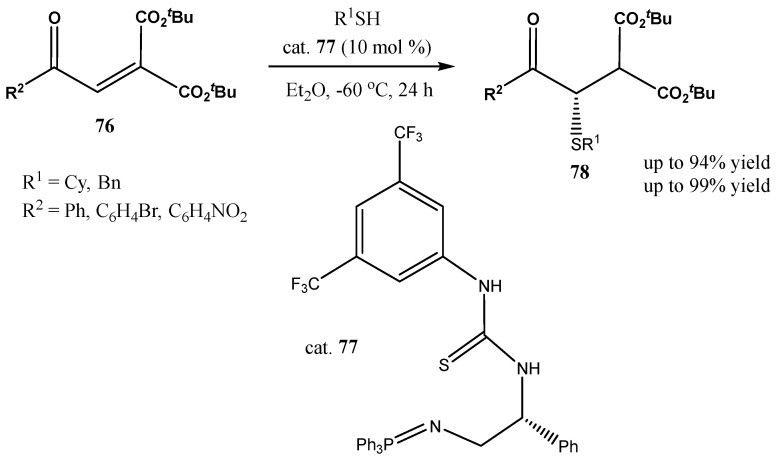
Asymmetric sulfa–Michael reaction of thiols with enone diesters.

Johnson and co-workers reported an asymmetric organocatalytic sulfa-Michael addition of thiols to highly activated enone diesters [99]. Employing a bifunctional cinchona alkaloid-derived catalyst **77**, a variety of aromatic and aliphatic thiols were added to enone diesters **76** with excellent stereocontrol, furnishing β-thioether products **78** bearing synthetically valuable 1,5-dicarbonyl frameworks (Scheme 23). The reaction proceeded in high yields and good to excellent enantioselectivities across a broad substrate scope. This study further expanded the range of Michael acceptors suitable for organocatalytic asymmetric C–S bond formation and provided access to versatile sulfur-containing building blocks for subsequent synthetic elaboration.

**Scheme 24:**
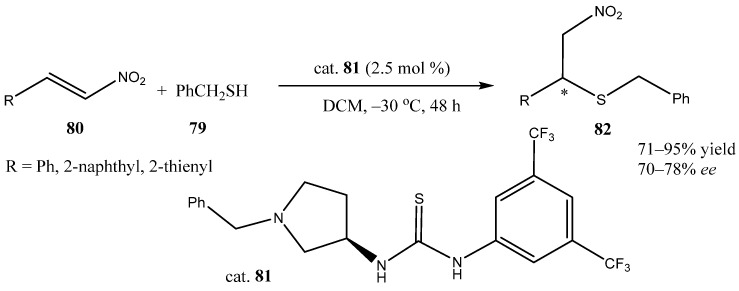
Asymmetric addition of thiol to nitro–olefins.

Skarżewski et al. reported an organocatalytic asymmetric addition of aliphatic thiols **79** to nitro olefins **80** and nitrodienes using cinchona alkaloid-derived catalysts **81** (Scheme 24) [100]. The methodology enabled efficient enantioselective C–S bond formation, providing β-nitro sulfides **82** in good yields and moderate to high enantioselectivities. The protocol was applicable to both nitroalkenes and conjugated nitrodienes.

### 4.2. Organocatalytic Sulfenylation Reactions

Organocatalytic asymmetric sulfenylation reactions represent an important class of enantioselective C–S bond-forming processes, enabling the direct introduction of sulfur-containing substituents into prochiral carbon frameworks under mild and metal-free conditions. In contrast to thio-Michael additions, these transformations generally proceed through the stereoselective transfer of an electrophilic sulfur species to an activated carbon nucleophile, most commonly generated via enamine or iminium activation of carbonyl compounds. Such reactions provide efficient access to α-sulfenylated aldehydes, ketones, and related derivatives, which constitute valuable intermediates in organic synthesis and medicinal chemistry [101]. The emergence of aminocatalysis has played a decisive role in the development of asymmetric sulfenylation methodologies. Chiral secondary amines derived from proline, imidazolidinones, and related scaffolds have been shown to effectively control both the reactivity and stereochemical outcome of electrophilic sulfur-transfer reactions. These catalytic systems have enabled the enantioselective construction of carbon stereocenters through direct C–S bond formation with a broad range of sulfur electrophiles and carbonyl substrates [101]. Subsequent developments expanded the scope of organocatalytic sulfenylation beyond simple aldehydes and ketones to encompass β-ketoesters, oxindoles, and other activated carbonyl compounds. As a result, asymmetric sulfenylation has evolved into a versatile synthetic strategy for the preparation of structurally diverse sulfur-containing molecules and remains an active area of research within organocatalytic asymmetric synthesis [102,103,104,105].

**Scheme 25:**
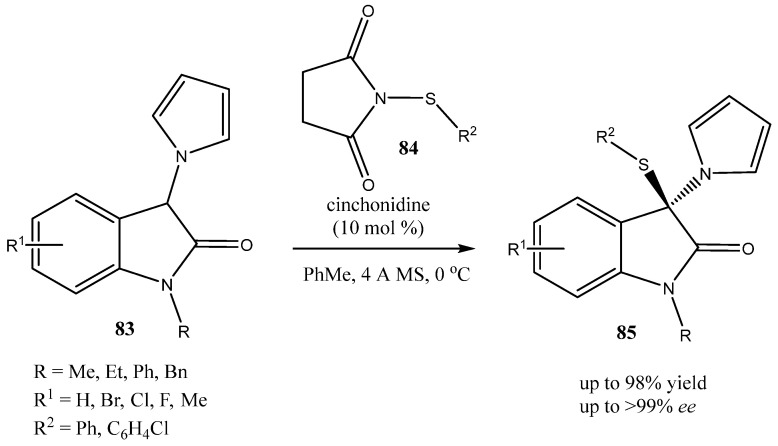
Asymmetric sulfenylation of 3-pyrrolyl-oxindoles.

Yuan et al. developed an organocatalytic asymmetric sulfenylation and selenenylation of 3-pyrrolyl-oxindoles **83** using electrophilic sulfur and selenium transfer reagents **84** [102]. Employing commercially available cinchonidine as catalyst, a broad range of 3-thio-3-pyrrolyl- and 3-seleno-3-pyrrolyl-oxindoles **85** bearing a quaternary stereogenic center at the C3 position were obtained in high yields and good to excellent enantioselectivities (Scheme 25). The methodology provided efficient access to 3,3-disubstituted oxindoles containing two different heteroatoms (N,S or N,Se) and was successfully demonstrated on gram scale.

**Scheme 26:**
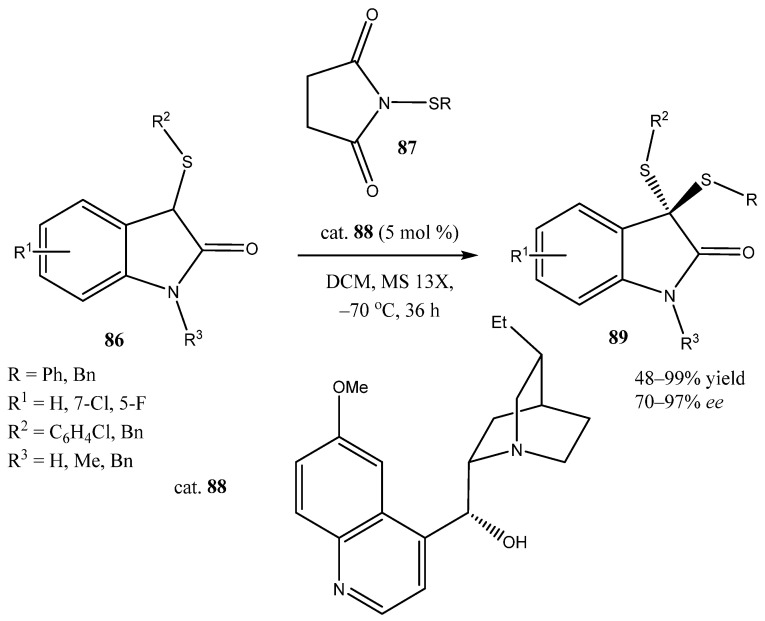
Catalytic asymmetric sulfenylation to dithioketals.

Liao and co-workers developed a catalytic asymmetric sulfenylation strategy for the synthesis of structurally diverse chiral dithioketals [103]. Using a cinchona alkaloid-derived organocatalyst **88**, a variety of sulfur-containing nucleophiles **86** were coupled with electrophilic sulfenylating reagents **87** through highly enantioselective C–S bond-forming processes (Scheme 26). The methodology provided access to a broad range of dithioketals **89** bearing sulfur-substituted stereogenic centers in high yields and excellent enantioselectivities. Importantly, the protocol demonstrated a rare example of organocatalytic asymmetric construction of sulfur-rich molecular architectures and significantly expanded the scope of organocatalytic sulfenylation chemistry beyond conventional α-sulfenylated carbonyl compounds.

**Scheme 27:**
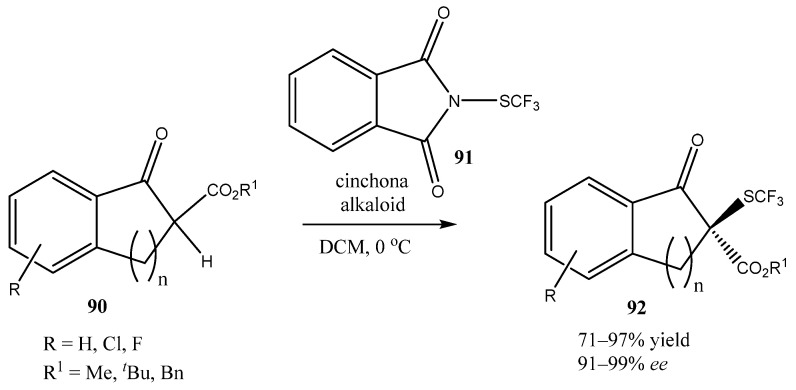
Asymmetric trifluoromethylsulfenylation.

In 2023, the authors developed an organocatalytic asymmetric trifluoromethylsulfenylation of β-ketoesters **90** using *N*-trifluoromethylthiophthalimide **91** as an electrophilic SCF_3_-transfer reagent [104]. Cinchona alkaloid-derived catalysts enabled highly enantioselective C–S bond formation, providing a range of α-trifluoromethylthio-substituted β-ketoesters **92** in high yields and good to excellent enantioselectivities (Scheme 27). The use of pseudoenantiomeric catalysts allowed access to both product enantiomers, and the synthetic utility of the obtained compounds was demonstrated through subsequent transformations into optically enriched α-SCF_3_ β-hydroxyesters.

**Scheme 28:**
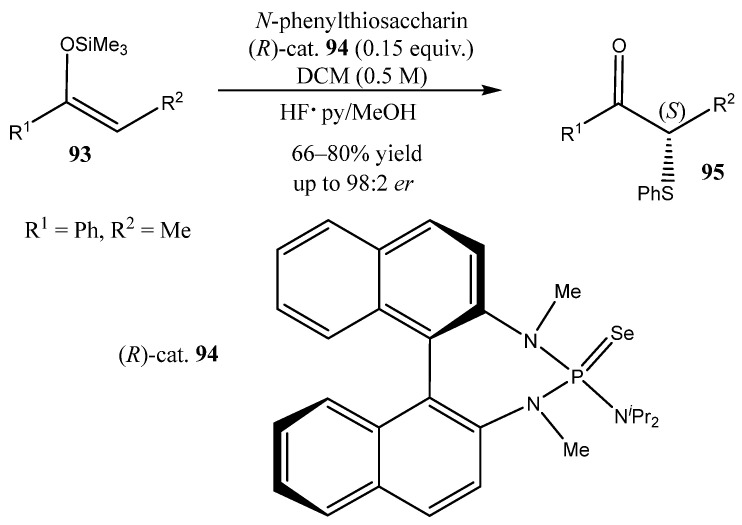
Enantioselective sulfenylation of ketone–derived enoxysilanes.

Denmark and co-workers developed a catalytic enantioselective α-sulfenylation of ketone-derived silyl enol ethers **93** using chiral Lewis base **94** catalysis [105]. A key feature of the methodology was the design of *N*-phenylthiosaccharin as a highly effective electrophilic sulfur-transfer reagent that avoided the need for Brønsted acid activation and prevented hydrolysis of the sensitive enoxysilane substrates. Chiral selenophosphoramides were identified as the most efficient catalysts, enabling the synthesis of α-phenylthio ketones **95** in high yields and good enantioselectivities (Scheme 28). Detailed computational studies further provided insight into the origin of stereocontrol in the sulfenylation process.

## 5. Emerging Photochemical and Electrochemical Approaches

The development of photochemical and electrochemical activation strategies has recently opened new opportunities for carbon–sulfur bond formation under mild and environmentally benign conditions. Unlike traditional ionic pathways commonly employed in transition-metal-catalyzed and organocatalytic transformations, these approaches frequently rely on the generation of sulfur-centered and carbon-centered radical intermediates, enabling access to distinct reactivity patterns and previously challenging bond-forming processes [106,107,108]. Visible-light photocatalysis has emerged as a particularly powerful platform for sulfur functionalization reactions, allowing the controlled generation of reactive sulfur species from thiols, disulfides, sulfinates, and related sulfur-containing precursors. As a result, numerous photocatalytic thiolation, sulfenylation, sulfonylation, and C–H functionalization reactions have been developed over the past decade [106,107,108]. Likewise, advances in synthetic organic electrochemistry have provided efficient methods for the generation of radical intermediates through direct anodic or cathodic activation, offering sustainable alternatives to conventional redox processes. Despite these significant advances, examples of enantioselective C–S bond-forming reactions employing photochemical or electrochemical activation remain remarkably scarce. The inherent difficulty of controlling stereochemistry in open-shell processes has long limited the development of asymmetric variants. Nevertheless, several recent studies have successfully combined radical generation with chiral catalytic systems, demonstrating that high levels of stereocontrol can be achieved in both photocatalytic and electrochemical C–S bond-forming transformations. These pioneering reports highlight the considerable potential of photochemical and electrochemical strategies for future developments in asymmetric organosulfur synthesis.

As already mentioned in Section 3.2, Wang and co-workers reported a copper-catalyzed asymmetric C–H sulfilimination of arenes via a HAT-initiated radical–radical coupling strategy [56]. The method relies on selective hydrogen atom transfer to generate carbon-centered radicals, which subsequently undergo stereocontrolled coupling with sulfur-centered radical intermediates in the presence of a chiral copper catalyst (Scheme 5). This approach enables direct enantioselective C–S bond formation from unactivated C–H bonds, providing a broad range of sulfilimines in high yields and excellent enantioselectivities. Mechanistic studies supported a radical pathway involving HAT activation and copper-controlled radical–radical coupling as the enantiodetermining step.

**Scheme 29:**
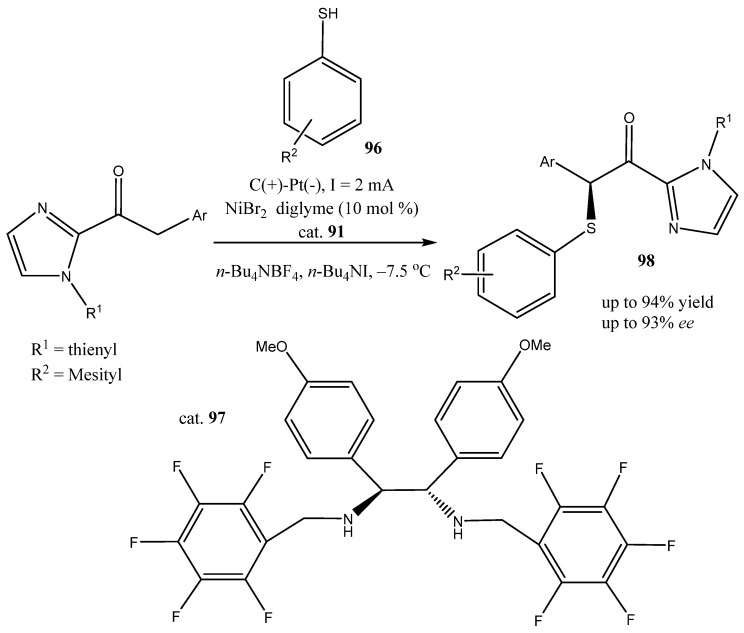
Asymmetric electrochemical C–S bond formation.

Sun and co-workers developed a nickel-catalyzed electrochemical strategy for the direct enantioselective construction of C–S bonds through radical cross-coupling [109]. Under electrochemical conditions, benzylic radicals generated by anodic oxidation were combined with sulfur-centered radicals derived from thiophenols **96** in the presence of a chiral nickel catalyst **97** (Scheme 29). The method provided a broad range of chiral thioethers **98** bearing stereogenic carbon centers in excellent yields and enantioselectivities of up to 93% *ee*. Mechanistic studies suggested that electrochemically generated carbon- and sulfur-centered radicals undergo nickel-controlled stereoselective coupling, highlighting the potential of asymmetric electrocatalysis for the synthesis of enantioenriched sulfur-containing compounds.

It should be also mentioned that some recent studies have demonstrated that radical processes involving sulfur dioxide can be rendered stereoselective through Curtin–Hammett-controlled pathways, illustrating the growing sophistication of radical sulfur chemistry [110,111].

## 6. Sulfur-Centered Stereogenicity Generated Through C–S Bond Formation

The rapidly expanding chemistry of sulfur-centered stereogenicity has been summarized in several recent reviews, including comprehensive discussions of asymmetric methods for the synthesis of S-stereogenic sulfoximines [112]. The asymmetric synthesis of sulfur-containing compounds has traditionally focused on the generation of stereogenic carbon centers through enantioselective C–S bond-forming reactions. In recent years, however, increasing attention has been directed toward the direct construction of sulfur-centered stereogenic units, reflecting the growing importance of chiral sulfur compounds in medicinal chemistry, asymmetric catalysis, and materials science. Sulfoxides, sulfoximines, sulfilimines, sulfinamides, and related sulfur(VI) and sulfur(IV) derivatives have emerged as valuable structural motifs owing to their unique stereochemical and physicochemical properties [56,113,114,115]. Among the various approaches to sulfur-centered chirality, asymmetric C–S bond formation represents an attractive strategy because it enables the simultaneous introduction of sulfur functionality and stereochemical information in a single catalytic transformation. Significant advances in transition-metal catalysis, organocatalysis, and radical-mediated processes have recently enabled the efficient preparation of sulfur-stereogenic compounds with high levels of enantiocontrol. In addition, several methodologies have demonstrated the simultaneous generation of sulfur-centered chirality and other stereochemical elements, including axial chirality and carbon-centered stereogenicity. These developments have substantially expanded the synthetic toolbox available for the preparation of enantioenriched sulfur-containing molecules and have established asymmetric C–S bond formation as a powerful platform for accessing architecturally complex sulfur-stereogenic frameworks.

**Scheme 30:**
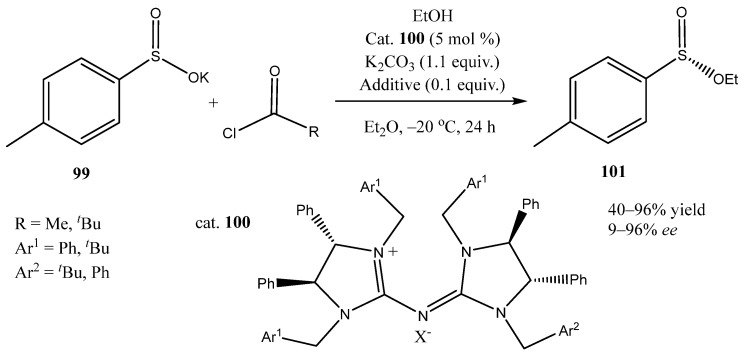
Asymmetric synthesis of sulfinate esters.

Zhang and co-workers reported a highly efficient strategy for the synthesis of sulfur-stereogenic sulfinate esters through a catalytic asymmetric condensation process [113]. The method relies on the enantioselective coupling of prochiral sulfinic acid derivatives **99** with alcohol nucleophiles under the control of a chiral organocatalyst **100**, enabling direct formation of the S–O bond while simultaneously establishing sulfur-centered chirality. A broad range of sulfinate esters **101** was obtained in excellent yields and with outstanding enantioselectivities, demonstrating broad substrate scope and high functional-group tolerance (Scheme 30). Mechanistic studies suggested that the catalyst-controlled condensation proceeds through selective activation and differentiation of the enantiotopic lone pairs at sulfur, providing an effective solution to the long-standing challenge of catalytic asymmetric synthesis of sulfur-stereogenic sulfinate esters. This work represents one of the first general catalytic approaches for the direct preparation of highly enantioenriched sulfinate esters and significantly expands the toolbox for sulfur-centered asymmetric synthesis.

**Scheme 31:**
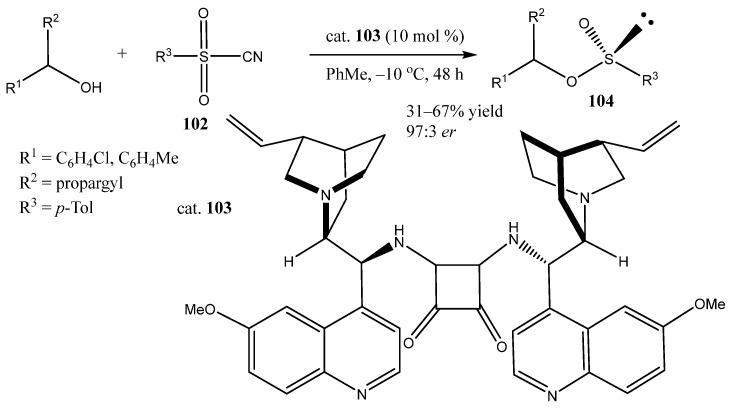
Asymmetric deoxygenation of sulfones.

Huang and co-workers developed an organocatalytic asymmetric deoxygenation of sulfones that provides direct access to sulfur-stereogenic sulfinyl compounds [114]. The strategy employs a chiral organocatalyst **103** to achieve selective reduction of prochiral sulfones **102**, enabling efficient differentiation of the enantiotopic oxygen atoms and generation of sulfur-centered chirality (Scheme 31). A broad range of sulfinyl products **104** was obtained in high yields and excellent enantioselectivities, demonstrating the generality of the methodology and its tolerance toward diverse functional groups. Mechanistic investigations suggested that catalyst-controlled deoxygenation constitutes the stereodetermining step, allowing precise control over the configuration of the sulfur stereocenter. This work represents a significant advance in asymmetric sulfur chemistry by providing a practical catalytic route to enantioenriched sulfinyl compounds from readily available sulfone precursors.

**Scheme 32:**
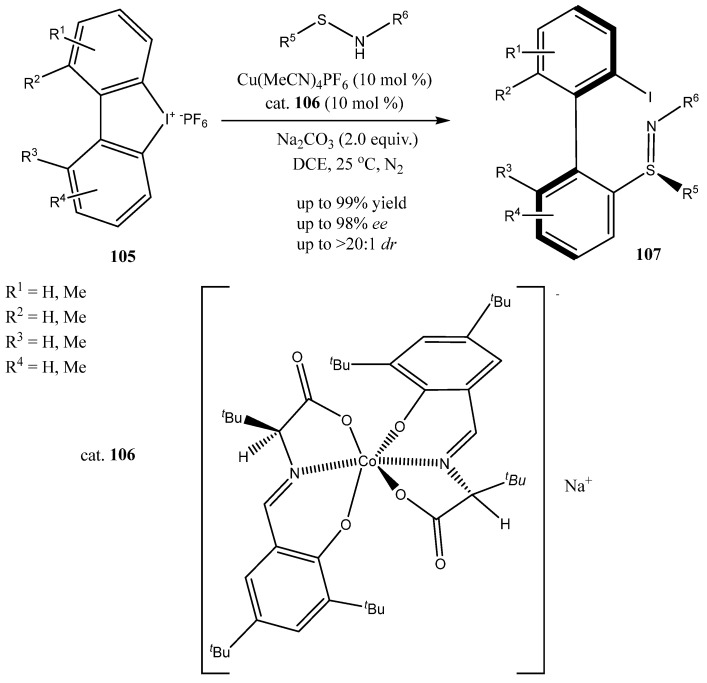
Asymmetric synthesis of biaryl sulfilimines.

Zhang et al. reported an asymmetric synthesis of biaryl sulfilimines **107** bearing multiple chiral elements through the reaction of sulfur-containing substrates with cyclic diaryliodonium salts **105** [115,116]. The methodology enabled the efficient construction of sulfur-stereogenic sulfilimines while simultaneously controlling additional stereochemical elements, including axial chirality in biaryl frameworks (Scheme 32). A broad range of products was obtained in the presence of catalyst **106** with high levels of enantio- and diastereoselectivity, demonstrating the effectiveness of the catalytic system in orchestrating multiple stereochemical features within a single transformation. The resulting sulfilimines could be further derivatized without erosion of stereochemical integrity, highlighting their synthetic utility.

**Scheme 33:**
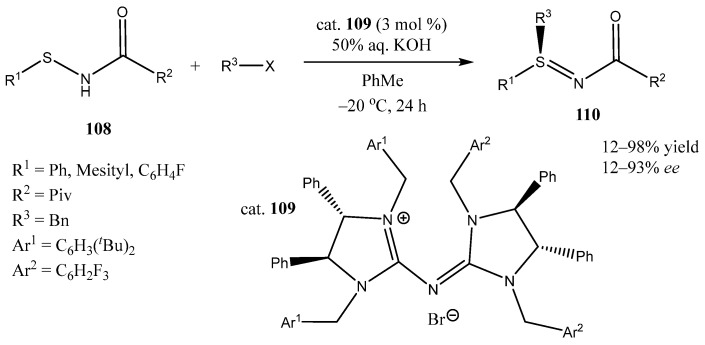
Enantioselective sulfur alkylation of sulfenamides.

In 2024, Wang and co-workers described an organocatalytic enantioselective sulfur alkylation of sulfenamides **108** for the synthesis of sulfur-stereogenic sulfilimines [117]. The strategy relies on catalyst-controlled (cat. **109**) differentiation of the enantiotopic lone pairs at sulfur, enabling direct formation of a stereogenic sulfur center during the alkylation process. A broad range of sulfilimines **110** was obtained in high yields and excellent enantioselectivities, demonstrating the versatility of the methodology and its broad substrate scope (Scheme 33). The resulting products could be readily transformed into structurally diverse sulfur-containing compounds while preserving stereochemical integrity.

**Scheme 34:**
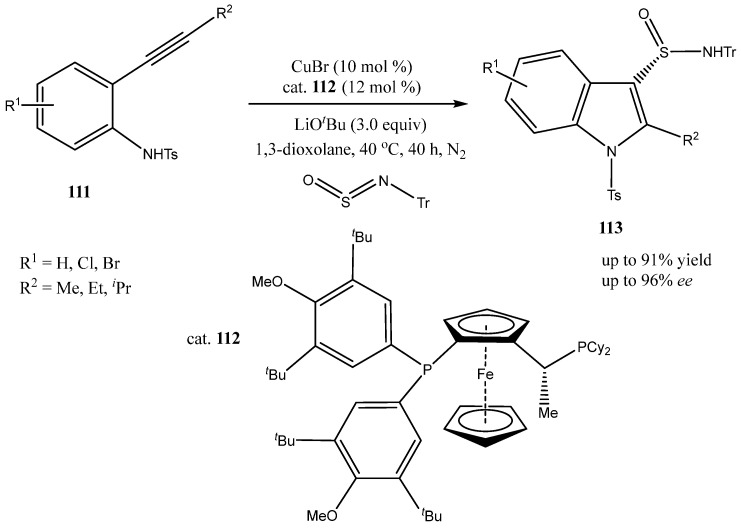
Asymmetric cyclizative sulfinamidation.

Shi and co-workers reported a similar catalytic asymmetric strategy for the synthesis of sulfur-stereogenic sulfilimines through direct S–C bond formation [118]. The methodology enables the construction of chiral sulfilimines via type **109** catalyst-controlled functionalization at sulfur, providing efficient access to a broad range of sulfur-stereogenic products with high yields and excellent enantioselectivities. The reaction exhibited broad substrate scope and excellent functional-group tolerance, highlighting its general applicability. Fang and co-workers developed a copper-catalyzed asymmetric cyclizative sulfinamidation of propargylic derivatives **111** that enables the efficient construction of indole-based sulfur-stereogenic sulfinamides **113** [58]. The transformation proceeds through an enantioselective intramolecular cyclization process, simultaneously generating a stereogenic sulfur(IV) center and axial chirality within the resulting heterocyclic framework. A broad range of indole-derived products **113** was obtained in high yields with excellent enantio- and diastereoselectivities, demonstrating the effectiveness of the chiral copper catalytic system **111** (Scheme 34). Notably, the methodology allows the concurrent control of sulfur-centered and axial chirality, providing access to architecturally complex sulfur-containing molecules.

**Scheme 35:**
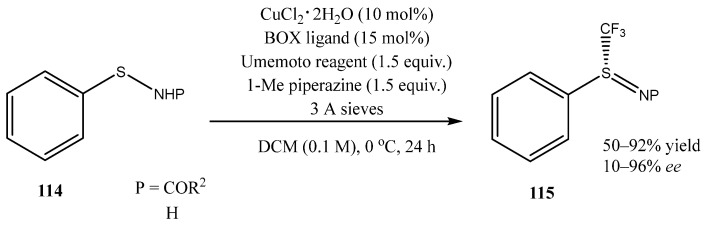
Asymmetric synthesis of sulfilimines.

Recently, Kwon, Ye and Ellman developed the first general catalytic enantioselective synthesis of S-trifluoromethyl sulfilimines through Cu(II)/BOX-catalyzed S-trifluoromethylation of sulfenamides **114** using the Umemoto reagent [119]. The method exhibited broad substrate scope, providing both S-(hetero)aryl and S-alkyl sulfilimines **115** in excellent yields and enantioselectivities (up to 99% yield and 99:1 *er*) (Scheme 35). Furthermore, the obtained sulfilimines served as versatile intermediates for the stereospecific synthesis of S-CF_3_ sulfoxides via hydrolysis with inversion of configuration and S-CF_3_ sulfoximines via stereoretentive oxidation, thereby establishing the first general asymmetric access to these pharmaceutically important sulfur-stereogenic motifs.

**Scheme 36:**
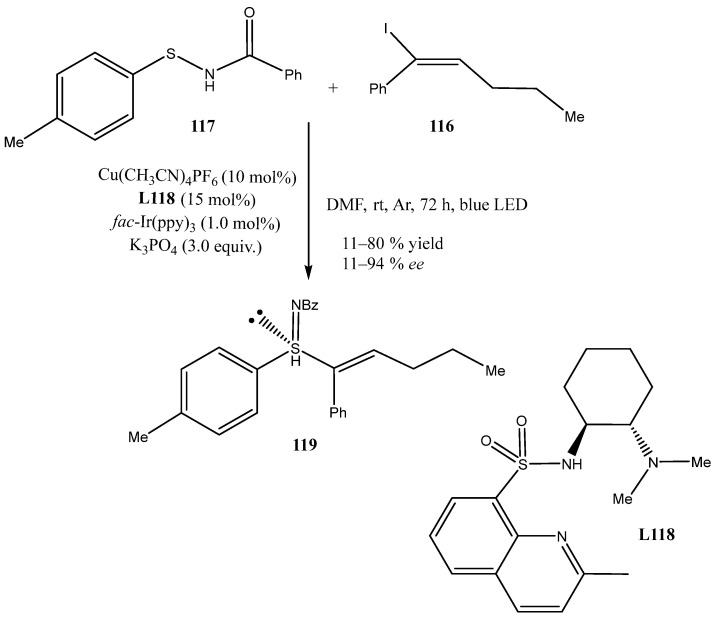
Synthesis of chiral sulfilimines from sulfenamides.

Very recently, Li and co-workers reported a copper/photoredox dual catalytic strategy for the enantioselective radical sulfilimination of alkenyl iodides **116** with sulfenamides **117** under action of the catalyst type **L118** (Scheme 36) [120]. Under visible-light irradiation, alkenyl radicals generated from alkenyl iodides underwent stereocontrolled coupling with chiral copper-bound sulfenamide intermediates, providing a broad range of sulfur-stereogenic alkenyl sulfilimines **119** in good yields and excellent enantioselectivities. The protocol exhibited high tolerance toward both aryl- and alkyl-substituted alkenyl iodides and could be further extended to radical 1,2-carbosulfilimination of alkynes, thereby considerably expanding the synthetic utility of asymmetric radical C–S bond formation.

**Scheme 37:**
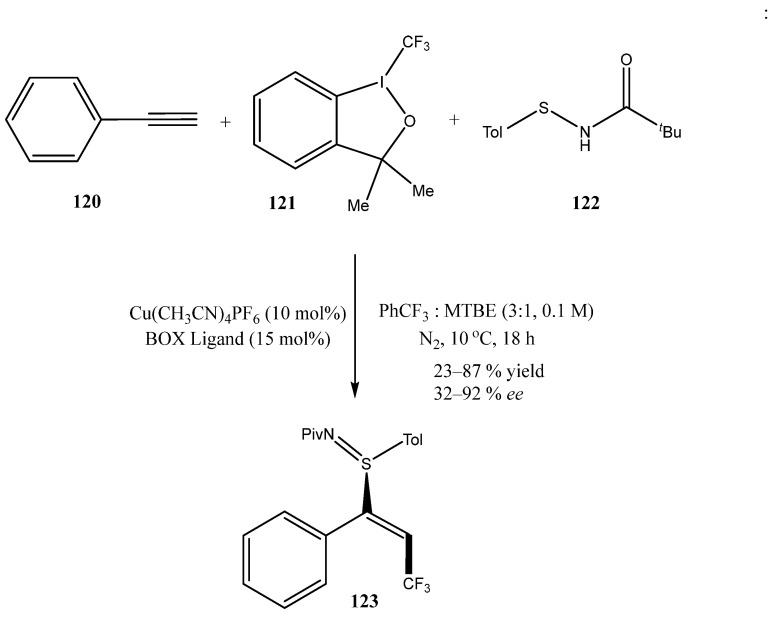
Trifluoromethyl-sulfilimination of terminal alkynes.

In another recent contribution, Li and co-workers developed a copper-catalyzed enantioselective radical relay trifluoromethyl-sulfilimination of terminal alkynes **120** in the presence of Togni-I reagent **121** and sulfenamide **122**, providing direct access to axially chiral vinyl sulfilimines **123** bearing sulfur-centered stereogenicity (Scheme 37) [121]. The transformation employs a Cu/photoredox dual catalytic system to promote radical trifluoromethylation followed by stereocontrolled C–S bond formation, delivering a wide range of vinyl sulfilimines in excellent yields with high enantio- and atroposelectivities. Mechanistic studies suggested a radical relay pathway involving CF_3_ radical addition to the alkyne and subsequent enantiodetermining capture of the vinyl radical by a chiral copper–sulfenamide complex. The resulting products could be readily transformed into structurally diverse sulfur(VI) compounds while preserving their stereochemical integrity, highlighting the synthetic potential of this strategy.

## 7. Mechanistic Aspects of Enantioinduction

**Figure 1:**
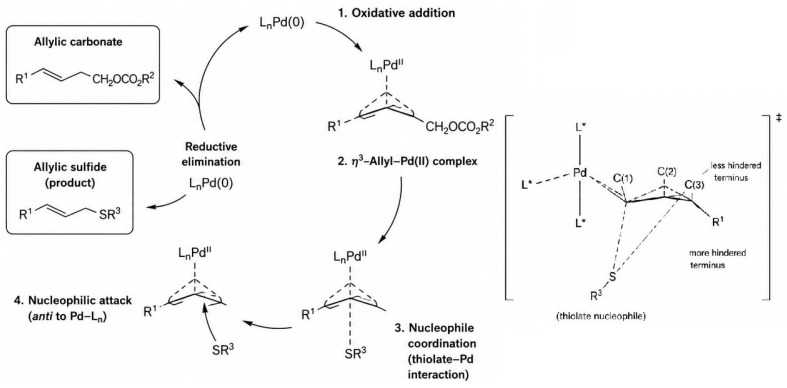
Generalized stereochemical model for Pd-catalyzed asymmetric π-allyl substitution with thiol nucleophiles.

**Figure 2:**
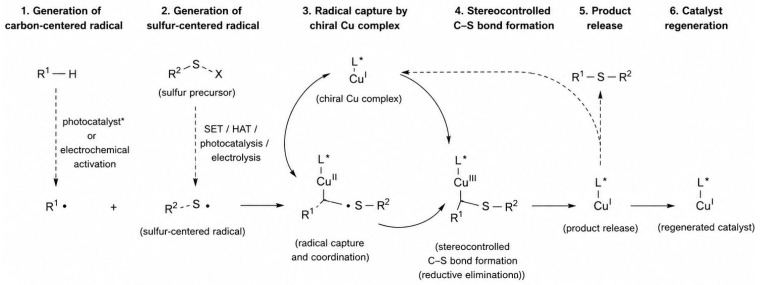
Generalized model for enantioselective radical C–S bond formation under chiral copper catalysis.

**Figure 3:**
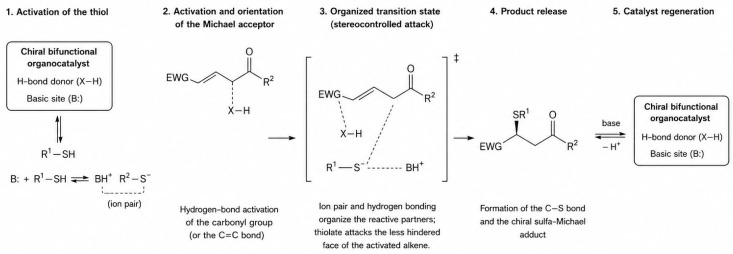
Generalized model of bifunctional organocatalytic activation in asymmetric sulfa-Michael addition.

Despite the structural diversity of asymmetric C–S bond-forming reactions, the underlying principles of enantioinduction are often remarkably similar. In most cases, stereochemical control is achieved through the creation of a well-defined chiral environment that selectively stabilizes one transition state over its competing enantiomeric counterpart. Depending on the catalytic platform employed, this control may arise from coordination to a chiral metal complex, non-covalent interactions in organocatalytic systems, or selective capture of reactive radical intermediates. In transition-metal-catalyzed reactions, enantioselectivity is typically governed by the spatial arrangement of substrates within the coordination sphere of the metal center. Chiral ligands create an asymmetric environment that differentiates between competing approaches of sulfur nucleophiles or electrophiles, thereby controlling the stereochemical outcome of the C–S bond-forming step. Such stereocontrol has been demonstrated in nickel-, copper-, rhodium-, iridium-, and palladium-catalyzed transformations, where the configuration of the product is established through catalyst-controlled migratory insertion, nucleophilic substitution, reductive elimination, or radical recombination processes. In organocatalytic reactions, stereochemical induction commonly relies on hydrogen bonding, ion pairing, or bifunctional activation of both reaction partners. Chiral thioureas, squaramides, cinchona alkaloid derivatives, and related catalysts organize the reacting substrates through non-covalent interactions, enabling highly selective sulfur additions to activated alkenes and carbonyl derivatives. The resulting transition-state organization allows efficient discrimination between the enantiotopic faces of prochiral substrates and provides high levels of enantiocontrol. More recently, photochemical and electrochemical approaches have introduced new challenges and opportunities for asymmetric C–S bond formation. In these systems, stereocontrol must be exerted over highly reactive open-shell intermediates generated through single-electron processes. Successful examples have demonstrated that chiral metal complexes can effectively intercept carbon- and sulfur-centered radicals, enabling enantioselective radical coupling and the construction of both carbon- and sulfur-centered stereogenic units. These advances illustrate the increasing ability of modern catalytic systems to achieve precise stereochemical control even in highly reactive radical environments. Overall, the development of asymmetric C–S bond-forming reactions highlights the importance of catalyst-controlled transition-state organization as a unifying principle of enantioinduction. Whether operating through ionic, coordination-driven, or radical pathways, contemporary catalytic systems rely on the selective stabilization of one stereochemical trajectory, thereby enabling efficient access to enantioenriched sulfur-containing molecules. As typical mechanistic examples, Pd-catalyzed π-allyl substitution with thiol nucleophile (Figure 1), Cu-catalyzed radical–radical coupling for C–S bond formation (Figure 2), and organocatalytic sulfa-Michael addition (Figure 3) were considered from the mechanistic point of view. In the first case, in a generalized mechanistic picture [122], the thiol nucleophile is activated by deprotonation and reacts with the chiral η^3^-allyl–Pd(II) intermediate. Depending on the catalytic system, thiolate formation and nucleophilic attack may involve different coordination and ion-pairing modes. The chiral ligand controls the spatial arrangement of the allyl fragment and nucleophile, thereby determining the preferred stereochemical pathway of C–S bond formation (Figure 1).

In the second case, in radical C–S bond-forming reactions, enantioinduction can arise from the controlled generation and capture of open-shell intermediates within a chiral copper environment [54]. Depending on the specific catalytic system, carbon- and sulfur-centered radical intermediates may undergo sequential or concerted interactions with copper complexes, ultimately leading to stereocontrolled C–S bond formation. Thus, the chiral metal complex can serve as a platform for controlling the relative orientation and recombination of highly reactive radical intermediates (Figure 2).

Finally, in bifunctional organocatalytic sulfa-Michael additions, enantioinduction generally results from the simultaneous organization of the Michael acceptor and activation of the thiol nucleophile through complementary noncovalent and acid–base interactions. Hydrogen bonding and/or ion-pairing interactions can orient the electrophilic alkene within the chiral environment of the catalyst, while the basic component promotes formation or stabilization of the reactive thiolate species. The resulting organized assembly directs C–S bond formation preferentially from one face of the activated alkene. The precise activation mode, however, depends on the structure of the catalyst and the specific Michael acceptor/nucleophile pair [123] (Figure 3).

## 8. Applications

The development of asymmetric C–S bond-forming reactions has significantly expanded the availability of enantioenriched organosulfur compounds, which occupy an important position in modern chemistry. Sulfur-containing molecules are widely encountered in pharmaceuticals, agrochemicals, natural products, functional materials, and chiral catalysts. Consequently, efficient methods for the stereoselective construction of C–S bonds have become indispensable tools for the preparation of structurally complex sulfur-containing compounds with precisely defined stereochemical properties. Particularly important are sulfur-stereogenic motifs such as sulfoxides, sulfoximines, sulfilimines, and sulfinamides, which have emerged as valuable structural elements in medicinal chemistry and asymmetric catalysis. In addition, numerous biologically active molecules contain sulfur-substituted stereogenic carbon centers that can be efficiently accessed through asymmetric thiolation and related C–S bond-forming reactions. The increasing availability of catalytic methods capable of generating both carbon- and sulfur-centered chirality has further broadened the utility of asymmetric sulfur chemistry in molecular design and synthesis. This section highlights representative applications of enantioenriched sulfur-containing compounds obtained through asymmetric C–S bond formation and illustrates their importance across diverse areas of contemporary chemical research.

### 8.1. Pharmaceuticals and Bioactive Molecules

**Figure 4:**
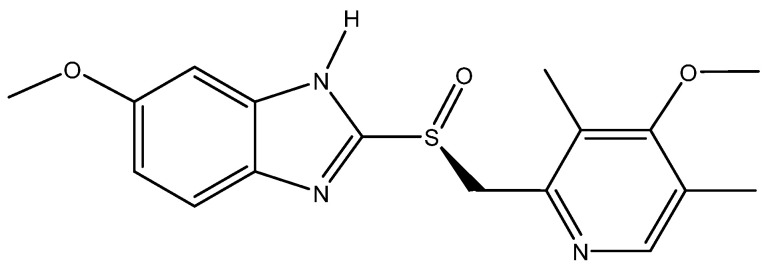
Structure of esomeprazole.

Sulfur-containing compounds occupy a prominent position in modern medicinal chemistry and are frequently encountered among approved drugs, clinical candidates, and biologically active natural products. The unique physicochemical properties of sulfur, including its variable oxidation states, polarizability, and ability to participate in diverse non-covalent interactions, often contribute significantly to biological activity and pharmacokinetic behavior. Consequently, sulfur-containing functional groups such as thioethers, sulfoxides, sulfones, sulfoximines, sulfilimines, and sulfonamides have become indispensable motifs in contemporary drug design [1,2,11]. Particularly important are chiral sulfur-containing molecules, as the absolute configuration of sulfur- or carbon-centered stereogenic units can strongly influence biological activity, metabolic stability, and target selectivity. A well-known example is esomeprazole, the single-enantiomer form of omeprazole, whose improved pharmacological profile highlights the importance of stereochemical control in sulfur-containing pharmaceuticals (Figure 4).

Similarly, increasing attention has been directed toward sulfur-stereogenic sulfoxides, sulfoximines, and related compounds, which are now recognized as valuable scaffolds in medicinal chemistry [11]. The growing demand for enantiomerically enriched organosulfur compounds has therefore stimulated the development of increasingly efficient catalytic methods for asymmetric C–S bond formation. These methodologies provide direct access to structurally diverse sulfur-containing building blocks and facilitate the synthesis of biologically relevant molecules that would otherwise require lengthy resolution or chiral auxiliary-based approaches. As a result, advances in asymmetric sulfur chemistry continue to play an important role in the discovery and development of new pharmaceutical agents.

### 8.2. Chiral Ligands and Catalysts

Beyond their importance as biologically active molecules, enantioenriched organosulfur compounds have found widespread applications as ligands and catalysts in asymmetric synthesis. Owing to the unique stereoelectronic properties of sulfur, chiral sulfur-containing ligands often provide highly efficient stereochemical control through a combination of electronic modulation, conformational rigidity, and well-defined three-dimensional architectures. In particular, sulfur-stereogenic sulfoxides have become one of the most extensively studied classes of chiral ligands for transition-metal-catalyzed asymmetric transformations [8,9]. Numerous sulfoxide-based ligands have been successfully employed in asymmetric hydrogenation, allylic substitution, conjugate addition, cycloaddition, and C–C bond-forming reactions, frequently providing excellent catalytic activity together with high levels of enantioselectivity. The development of chiral sulfoxide ligands has also provided an important alternative strategy for asymmetric allylic substitution. Recent advances in this area have demonstrated the ability of sulfoxide-based ligands to efficiently transfer stereochemical information to transition-metal centers, with applications spanning Pd-, Ru-, and Rh-catalyzed allylic substitution [124]. The stereogenic sulfur atom plays a decisive role in controlling the geometry of the metal coordination sphere and, consequently, the stereochemical outcome of the catalytic process. In addition to sulfoxides, sulfur-containing motifs such as sulfoximines and related sulfur(VI) compounds have recently attracted increasing attention as versatile chiral scaffolds for ligand design owing to their enhanced structural diversity and tunable steric and electronic properties [9,11]. The continuous development of asymmetric C–S bond-forming methodologies has significantly facilitated access to these valuable chiral sulfur-containing architectures. Consequently, modern catalytic approaches not only enable the synthesis of biologically relevant organosulfur compounds but also provide efficient routes to advanced ligands and catalysts that further expand the scope of asymmetric synthesis.

### 8.3. Functional Materials and Emerging Applications

In addition to their well-established roles in medicinal chemistry and asymmetric catalysis, chiral organosulfur compounds are attracting increasing attention as functional molecular building blocks. The unique stereoelectronic properties of sulfur, together with the availability of multiple oxidation states and tunable three-dimensional architectures, make sulfur-containing molecules attractive candidates for applications in molecular recognition, supramolecular chemistry, and functional materials [9,11]. Sulfur-stereogenic compounds such as sulfoxides, sulfoximines, and sulfilimines possess well-defined chiral environments and distinctive electronic characteristics that can be exploited in the design of chiral molecular receptors, optically active materials, and advanced functional architectures. Furthermore, the ability to precisely control both carbon- and sulfur-centered stereochemistry through modern asymmetric C–S bond-forming methodologies enables the preparation of increasingly complex molecular frameworks with potential applications in materials science and molecular engineering. Although these applications remain less developed than those in pharmaceutical chemistry, recent advances in catalytic asymmetric C–S bond formation have substantially expanded the structural diversity of accessible sulfur-containing compounds. Continued progress in catalyst design and stereoselective synthesis is therefore expected to facilitate the development of new functional materials and broaden the impact of sulfur-centered chirality beyond traditional synthetic and medicinal chemistry.

## 9. Conclusions and Outlook

Over the past three decades, asymmetric C–S bond formation has evolved from a relatively underdeveloped area into a versatile and rapidly expanding field of asymmetric synthesis. Significant advances in transition-metal catalysis, organocatalysis, and, more recently, radical-based catalytic strategies have enabled the efficient construction of sulfur-containing molecules bearing both carbon- and sulfur-centered stereogenic elements. The continuous development of highly efficient catalytic systems has substantially broadened the substrate scope, improved functional-group tolerance, and provided access to increasingly complex organosulfur architectures with excellent levels of enantioselectivity. Among the various catalytic platforms, palladium-, copper-, rhodium-, nickel-, and iridium-catalyzed methodologies have established reliable strategies for asymmetric C–S bond construction through diverse reaction pathways, including allylic substitution, conjugate addition, hydrogenation, C–H functionalization, and radical coupling. In parallel, organocatalytic approaches have demonstrated remarkable efficiency in asymmetric sulfa-Michael additions and electrophilic sulfenylation reactions by exploiting hydrogen-bonding and bifunctional activation modes. More recently, isolated examples of asymmetric photocatalytic and electrochemical C–S bond-forming reactions have illustrated the feasibility of extending stereocontrol to single-electron processes, opening new opportunities for catalyst design. Despite these impressive achievements, several important challenges remain. Many catalytic systems still exhibit limited substrate generality, often requiring carefully tailored substrates or highly specific sulfur coupling partners. Enantioselective construction of sulfur-centered stereogenic compounds remains considerably less developed than the synthesis of carbon-centered stereocenters, and general catalytic strategies applicable to a broad range of sulfur oxidation states are still scarce. Furthermore, many currently available methods rely on expensive transition metals, specialized chiral ligands, or elaborate catalyst preparation, which may restrict their practical application on larger scales. Another significant limitation is the relatively narrow diversity of reaction classes currently available for asymmetric C–S bond formation. While asymmetric allylic substitutions and sulfa-Michael additions are now well established, comparatively few catalytic systems have been developed for enantioselective cross-coupling reactions, direct C–H thiolation, radical C–S bond formation, or electrochemical transformations. Similarly, asymmetric methodologies capable of simultaneously controlling multiple stereochemical elements, including sulfur-centered chirality, axial chirality, and central chirality, are only beginning to emerge. Future research will likely focus on the development of more sustainable and broadly applicable catalytic systems. In particular, the replacement of precious metals with earth-abundant catalysts, the expansion of asymmetric photochemical and electrochemical methodologies, and the integration of dual catalytic strategies represent promising directions for future investigation. The increasing use of computational chemistry, mechanistic studies, and data-driven catalyst design is also expected to accelerate the discovery of more efficient catalytic platforms and improve the rational design of enantioselective C–S bond-forming reactions. Another particularly attractive direction involves the synthesis of sulfur-stereogenic compounds through direct catalytic C–S bond formation. Recent advances in the preparation of chiral sulfilimines, sulfinamides, sulfinate esters, and related sulfur(VI) compounds demonstrate that precise stereochemical control at sulfur is becoming increasingly achievable. Continued development of these methodologies is expected to provide access to new classes of biologically active molecules, chiral ligands, and functional materials. Overall, asymmetric C–S bond formation has matured into an indispensable component of modern synthetic chemistry. Although substantial challenges remain, the rapid emergence of innovative catalytic concepts and the growing demand for enantioenriched organosulfur compounds strongly suggest that this field will continue to expand. Future advances are expected not only to broaden the synthetic toolbox available to chemists but also to facilitate the discovery of new pharmaceuticals, catalysts, and functional materials based on precisely controlled sulfur stereochemistry.

## Figures and Tables

**Table 1:** Classification of catalytic enantioselective C–S bond forming reactions.

Strategy	Catalyst Type	Typical Products
Allylic substitution	Pd, Ir	Allylic sulfides
Conjugate addition	Cu, Rh	B-Thio carbonyls
Cross-coupling C-H functionalization Organocatalytic sulfenylation	Cu, Ni, Pd Rh, Co, Pd CPA, thiourea, squaramide	Aryl and alkyl sulfides Functionalized sulfides Chiral sulfides
Photoredox catalysis	Dual catalysis	Radical-derived sulfides
Electrocatalysis Sulfur-centered stereogenicity generation	Chiral electrocatalytic systems Various systems	Sulfenylated products Sulfoximines, sulfilimines, sulfinamides

## Data Availability

No new data were created or analyzed in this study. Data sharing is not applicable to this article.
